# Supervised learning model predicts protein adsorption to carbon nanotubes

**DOI:** 10.1126/sciadv.abm0898

**Published:** 2022-01-07

**Authors:** Nicholas Ouassil, Rebecca L. Pinals, Jackson Travis Del Bonis-O’Donnell, Jeffrey W. Wang, Markita P. Landry

**Affiliations:** 1 Department of Chemical and Biomolecular Engineering, University of California, Berkeley, Berkeley, CA 94720, USA.; 2 Picower Institute for Learning and Memory, Massachusetts Institute of Technology, Cambridge, MA 02139, USA.; 3 Chan Zuckerberg Biohub, San Francisco, CA 94158, USA.; 4 Innovative Genomics Institute (IGI), Berkeley, CA 94720, USA.; 5 California Institute for Quantitative Biosciences (QB3), University of California, Berkeley, Berkeley, CA 94720, USA.

## Abstract

Engineered nanoparticles are advantageous for biotechnology applications including biomolecular sensing and delivery. However, testing compatibility and function of nanotechnologies in biological systems requires a heuristic approach, where unpredictable protein corona formation prevents their effective implementation. We develop a random forest classifier trained with mass spectrometry data to identify proteins that adsorb to nanoparticles based solely on the protein sequence (78% accuracy, 70% precision). We model proteins that populate the corona of a single-walled carbon nanotube (SWCNT)–based nanosensor and study the relationship between the protein’s amino acid–based properties and binding capacity. Protein features associated with increased likelihood of SWCNT binding include high content of solvent-exposed glycines and nonsecondary structure–associated amino acids. To evaluate its predictive power, we apply the classifier to identify proteins with high binding affinity to SWCNTs, with experimental validation. The developed classifier provides a step toward undertaking the otherwise intractable problem of predicting protein-nanoparticle interactions.

## Teaser

The developed classifier predicts what proteins adsorb to nanoparticles and what protein features drive these interactions.

## INTRODUCTION

Engineered nanoparticles are poised to transform how we undertake biological sensing (*1*, *2*), imaging (*3*, *4*), and delivery (*5*–*7*): Nanoscale materials enable localization within otherwise inaccessible biological environments and exhibit highly tunable physicochemical properties to tailor function. Different nanoparticle platforms offer application-dependent advantages, such as near-infrared fluorescent nanoparticles for through-tissue imaging (*8*, *9*) or biodegradable nanoparticles for in vivo delivery (*10*–*12*). In particular, single-walled carbon nanotubes (SWCNTs) are well suited for biological sensing and imaging due to their tissue-transparent and photostable near-infrared fluorescence, in addition to their readily modifiable surface (*13*–*15*). Accordingly, SWCNTs have been functionalized with biomolecules including single-stranded DNA (ssDNA) to create neurotransmitter nanosensors (*16*–*18*), with peptide mimetics to form protein nanosensors (*19*), and with proteins to construct viral nanosensors (*20*). Similarly, the large SWCNT surface area enables cargo attachment such that SWCNTs can be loaded with DNA plasmids or small interfering RNAs, translocating these functional biomolecules into cells for gene expression and silencing applications (*21*, *22*). Optimizing these biomolecule-nanoparticle interactions is key in enhancing nanotechnology function, and a deeper understanding of these interfacial interactions would enable more rational conjugate designs. Hence, the capability to predict nano-bio interactions would aid the design phase of nanobiotechnologies by lessening the need to experimentally test innate interactions of each biomolecule with each nanoparticle of interest.

Although such aforementioned nano-bio interactions are required for function, conversely, biofouling resulting from undesired nano-bio interactions inhibits intended nanoparticle outcomes. SWCNTs and other nanotechnologies more broadly suffer from as-of-yet unpredictable interactions with the biological environments in which they are applied. When engineered nanoparticles are introduced into biological systems, endogenous proteins rapidly bind to the nanoparticle surface (*23*–*25*). This phenomenon is known as protein corona formation. Protein adsorption often decreases the ability of the nanoparticle to interact with its surrounding environment, such as sensing nearby analytes (*26*–*28*) or navigating biological barriers (*29*, *30*). Because of its inherent complexity, the protein corona remains a poorly understood phenomenon, limiting the efficiency with which nanoparticle-based technologies are applied in biological systems (*29*, *31*, *32*). Limitations in our understanding of corona formation arise from a convolution of diverse nanoparticle properties (dominated by surface characteristics) and the complexity of biological environments (*24*, *29*, *33*, *34*). Yet, knowledge of the proteins adsorbed in this corona phase would enable better prediction of the biological identity, and thus fate, of the applied nanotechnologies (*35*, *36*). Experimental testing to fully characterize the protein corona on all synthesized nanoparticle constructs within all intended biological environments is laborious and costly: While recent work has made headway toward high-throughput experimental methods (*37*, *38*), the most strategies rely on labor-intensive mass spectrometry (MS)–based proteomics (*33*, *39*, *40*). The ability to predict the protein corona that will form on nanoparticles in vivo remains a challenge that, if overcome, would improve applied nanotechnology performance.

Pattern recognition techniques, including those of machine learning, offer a route to characterize protein-nanoparticle interactions in a high-throughput manner across this extensive design space of nanoparticles applied in different biological systems. Previous work pioneering this idea applied random forest classification to predict proteins that adsorb to silver nanoparticles in biologically relevant environments (*39*) and has been expanded to larger nanoparticle libraries (*41*). However, certain aspects stand to be refined, such as setting the threshold of whether a protein is classified as in or out of the corona, and more broadly implementing these strategies to non-spherical nanoparticles. Other work has examined protein-nanoparticle complexes using a fluorometric assay to guide prediction of corona formation, although issues arise in characterizing graphene-based substrates (*42*). More broadly, most predictive modeling efforts involving nanoparticles applied in biology consider cellular- or organism-level responses, such as cellular association (*40*, *43*), toxicity (*44*), in vivo fate (*36*), and therapeutic efficacy (*43*, *45*). Toward protein-SWCNT conjugate design, some predictive modeling has informed protein candidates that exhibit a natural affinity for the graphitic SWCNT surface (*46*). For example, Di Giosia *et al.* (*47*) implemented a molecular docking model to determine a panel of proteins that interact with the carbon nanotube surface. Yet, this strategy of predicting protein corona identity requires protein structural information and is low throughput, both computationally and in experimental validation. Our workflow expands on this body work by classifying protein attachment to SWCNTs based only on protein sequence, as well as redefining metrics for determining in-corona placement.

Here, we develop a classifier to investigate the relationship between a protein’s amino acid sequence and a protein’s binding propensity to carbon nanotubes. Our purpose is twofold: As one objective, we aim to predict which protein-SWCNT interactions to expect in biological environments. This knowledge will inform implementation of anti-biofouling strategies toward effective biological application of nanoparticles. Our second objective is to predict high-affinity protein binders to SWCNTs and protein features associated with such binding affinity to improve the process of protein-nanoparticle construct design (*46*). Toward these ends, we build and optimize a random forest classifier (RFC) applied to protein adsorption on SWCNTs. We relate protein properties (derived from protein sequence data) to whether proteins are in or out of the corona phase on SWCNTs (experimentally determined by quantitative MS-based proteomics). Specifically, we focus on protein corona formation on (GT)_15_-SWCNTs due to their demonstrated applicability for dopamine sensing (*16*, *17*); however, the workflow is generalizable to other nanoparticles, as we briefly demonstrate with polystyrene nanoparticles (PNPs). We train our classifier using MS-based proteomic data characterizing the corona formed on (GT)_15_-SWCNTs in two relevant bioenvironments: the intravenous environment (blood plasma) and the brain environment [cerebrospinal fluid (CSF)] (*48*). We find that our classifier can precisely target the small number of proteins that adsorb to our nanoparticle. Furthermore, we identify population distribution changes among the most important protein properties to gain insight on how our classifier distinguishes positive targets. Namely, high content of glycine residues (particularly solvent-exposed residues) and amino acids not associated with secondary structure domains (not α helix, β sheet, or turns) leads to favorable SWCNT binding, whereas high content of leucine residues and amino acids associated with planar β-sheet domains leads to unfavorable SWCNT binding. These results imply that more conformationally flexible proteins can adapt to the highly curved SWCNT surface and maximize favorable surface contacts, while more internally stable proteins are less likely to reorient and bind to the nanotube surface. Last, we test our model with a new set of proteins and perform quantitative protein adsorption experiments to validate the model’s in versus out of corona predictions (*28*). Our results suggest that this classifier can serve as a tool to understand how protein sequence influences nanotube binding.

## RESULTS

### Experimentally determined protein corona composition on (GT)_15_-SWCNTs

The protein corona dataset was experimentally generated from a selective adsorption assay that quantified protein amounts present on nanoparticles via liquid chromatography–tandem MS (LC-MS/MS) characterization (*48*). With this assay, corona proteins were determined for (GT)_15_-SWCNTs incubated in either human blood plasma or CSF of the brain [attached datasheet, reproduced from (*48*)]. The absolute protein abundance and relative enrichment or depletion (compared to the control sample of the biofluid alone) were used to indicate whether a particular protein was considered to be in the corona, as will be described in a later section. We included four protein corona datasets: (GT)_15_-SWCNTs in blood plasma, (GT)_15_-SWCNTs in CSF, the total set with biofluid labels, and the total set naïve of biofluid labels. The biofluid label refers to the knowledge of where the protein originated (blood plasma or CSF).

### Protein property database development from protein sequence

We next curated a protein property database to use with our classifier. We used the amino acid sequence of each protein from the annotated protein database, UniProt (*49*), to construct an array of predicted physicochemical protein properties with the BioPython package (table S1; see the “Database development” section in Materials and Methods) (*50*). UniProt also provides biological protein properties such as gene ontology, sequence annotations, and specific functional regions; therefore, we compared how the inclusion of these other properties influenced classifier performance (fig. S1). Specifically, we hypothesized that proteins with relevant binding domains (such as for DNA) or relevant biological functions (such as binding and stabilizing hydrophobic molecules, e.g., lipids) may preferentially associate with the ssDNA-functionalized SWCNT surface. Yet, inclusion of such biological protein properties resulted in only minimal improvements to the preliminary classifier’s ability: The classifier with all protein features had an accuracy of 0.766 (compared to 0.760), area under the receiver operating curve (AUC) of 0.741 (compared to 0.734), precision of 0.690 (compared to 0.676), and recall of 0.585 (compared to 0.590). Therefore, our final classifier was based solely on amino acid sequence data due to only marginal performance increase with these added protein properties and to avoid the issue of less well-studied proteins with no empirically derived properties and/or no annotated features. Thus, using only the protein’s amino acid sequence enables facile expansion to model future experimental datasets and to select previously unidentified nanoparticle-binding proteins of interest.

The amino acid sequence of a protein provides valuable information including the percentage of a specific amino acid within the full protein; however, spatial organization is disregarded. To complement the sequence-derived dataset, we added the parameter of solvent accessibility that estimates the exposed protein surface area. We implemented NetSurfP 2.0 (*51*) to predict the number of exposed residues of a particular protein using the amino acid sequence, normalized by either the total number of amino acids or the total number of exposed amino acids. These two choices of normalization provide information on the corona-enriched proteins’ amino acid content on the surface relative to the full protein or relative to only other surface-exposed residues, respectively.

### Thresholding to determine protein placement: In or out of the corona

The decision of whether a protein was categorized as in or out of the corona was made using the protein abundance data from LC-MS/MS experiments. Proteins were placed into the corona based on two criteria: (i) relative change and (ii) an abundance threshold. First, if a protein was more abundant in the nanoparticle-bound case than it was in the control solution of the native biofluid without any nanoparticles present (i.e., enrichment on the nanoparticle), then it was classified as in the corona. Second, the remaining proteins were ordered by abundance and fit to an exponential distribution. Increasing the power of the exponential leads to a higher in-corona threshold, placing fewer proteins in the corona. This thresholding approach reflects that lower abundance of a protein in the corona relative to its abundance in the biofluid (i.e., depletion on the nanoparticle) does not necessarily mean that protein is out of the corona; a protein that is substantially depleted can still be present in the corona with a high absolute quantity. The thresholding method that we have developed is discussed further in Materials and Methods.

### RFC development and verification using established protein property database

We implemented an RFC to classify corona proteins on (GT)_15_-SWCNT nanoparticles. Although we initially focus on protein corona characterization with one nanoparticle type, SWCNTs, these classifiers do not require any information regarding the nanoparticle itself. We chose to pursue an RFC because this is an ensemble method with a well-known ability to be resistant to overfitting by using several weak learners that fit to different parameters (*52*). Moreover, an RFC produces highly interpretable results. Implementing an RFC is also in line with previously published work (*39*, *41*). An example tree to illustrate this process is provided in fig. S2. To confirm the choice of an RFC over other potential classifiers, we tested an assortment of classifier types (fig. S3). The highest performing classifiers were the RFC (with either 100 or 1000 trees) and XGBoost using decision trees, based on a sum of the metrics of accuracy, precision, and recall. We selected the RFC for this study because the accuracy (0.760) and precision (0.683) values were superior to that of the XGBoost decision tree while retaining a similar AUC (0.726). AUC is a frequently used measure for understanding sensitivity and specificity of the classifier. The high precision of the RFC (positive predictive value) is favorable for the most straightforward application of classifier output for nanobiotechnology optimization: More precise results are better for experimenters using this tool to correctly identify formerly unknown nanoparticle-binding proteins. However, the XGBoost classifier did perform better than the RFC in recall (XGBoost, 0.597; RFC, 0.583). Higher recall results are preferred when the opportunity cost of missing a positive corona contributor is more problematic than including a false positive.

**Fig. 1.**
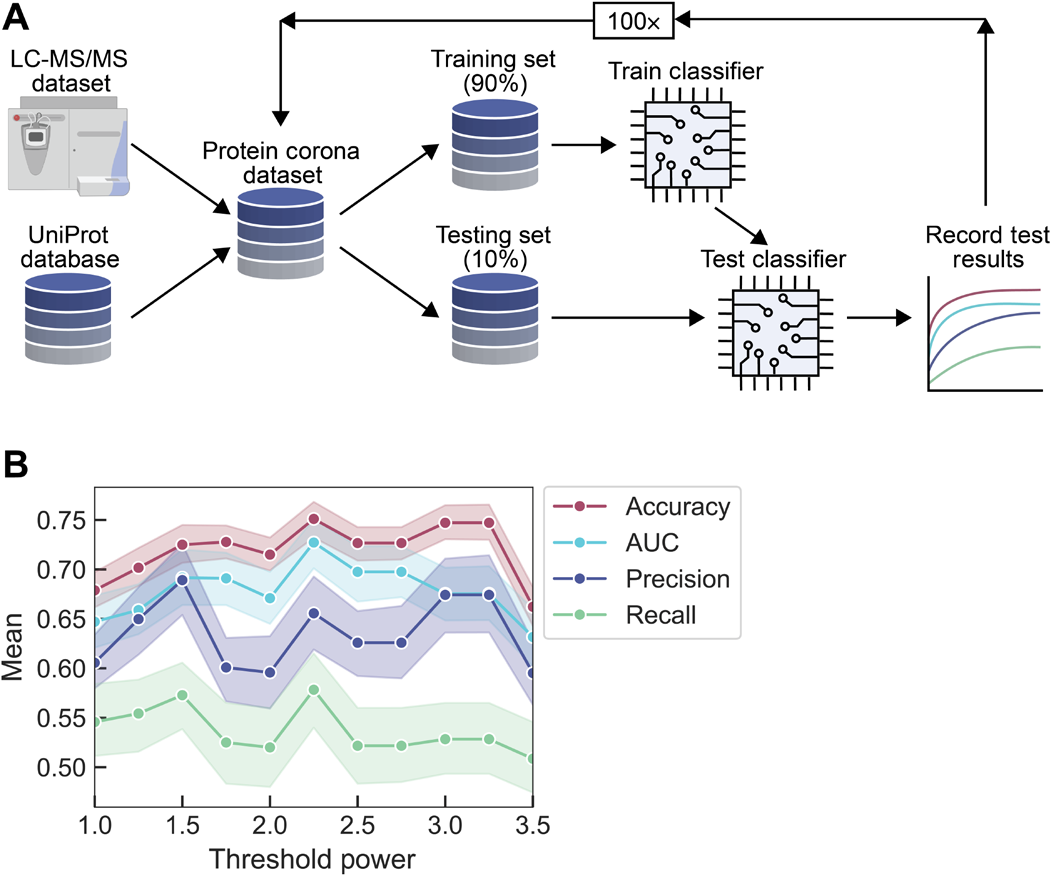
RFC workflow and development for determining proteins in versus out of the corona phase on (GT)_15_-SWCNTs. (**A**) RFC workflow used in splitting-based predictions. LC-MS/MS experimentally provides protein corona composition. LC-MS/MS data are combined with protein properties derived from the protein sequence (UniProt database with BioPython package for analysis) to form a total dataset. The total dataset is split 90% into training data and 10% into test data. Training data are used to train a reset classifier, and then test data are used to score the trained classifier. Results are recorded, and the process is repeated. (**B**) Metrics of accuracy, AUC, precision, and recall are recorded as a function of threshold power for labeling proteins as in versus out of the corona. A threshold power value of 2.25 is selected for subsequent analyses due to the optimal combination of the recorded metrics. Shaded error bars represent 95% confidence intervals.

During development, stratified shuffle split validation was used to check the success of our classifier with respect to accuracy, AUC, precision, and recall. The dataset was divided into a training and test set at the beginning of each split, and then the training set was fit to an untrained classifier. Next, predictions were made on the test set and compared with our true answers. The results from this classifier were saved, and the process was repeated with the classifier naïve at the beginning of each iteration, as graphically depicted in Fig. 1A. This method ensures that each protein revolved into the test set during one of the folds. Statistics represented in this work were generated from the *n* trials used in this verification step.

Using an RFC, classification tests were run on the total naïve dataset of proteins marked as being in or out of the corona with the aforementioned thresholding method. The classifier performance was scored for a range of thresholding powers (Fig. 1B). The classifier was then refreshed, and the standard protocol for training the classifier was repeated to gather metrics related to classification: accuracy, AUC, precision, and recall. The metrics were recorded until a thresholding power of 3.5, at which point higher powers considerably reduced the number of proteins counted in the corona and many metrics markedly declined in their performance. We ultimately selected a power of 2.25 because this power provided the best compromise between accuracy (0.751), AUC (0.727), precision (0.656), and recall (0.578). All reported results for the remainder of this work use a power of 2.25 for placing proteins in the nanoparticle corona.

To reconcile the imbalance in our LC-MS/MS experimental dataset (i.e., unequal number of proteins in either class), we up-sampled our minority class (in corona; ~30% in corona without up-sampling in the total dataset). This up-sampling ensures that each time the classifier was trained, we were able to recover an appropriate amount of the minority class. For this reason, the classifier was validated using a stratified shuffle split repeated 100 times. Moreover, we noticed that generalization of this classifier could be improved, especially when considering that the recall was below 0.6. To address this issue, a synthetic minority oversampling technique (SMOTE) (*53*) was implemented to generate “proteins” in the minority class (in corona). This analysis revealed that the most accurate and precise results for our classifier were obtained when the minority/majority ratio in SMOTE was 0.7:1.0 (fig. S4), with substantially improved recall from 0.583 to 0.647. Introducing the described methods expanded the number of proteins that were placed in the corona and accordingly enhanced the predictive power of the classifier.

**Fig. 2.**
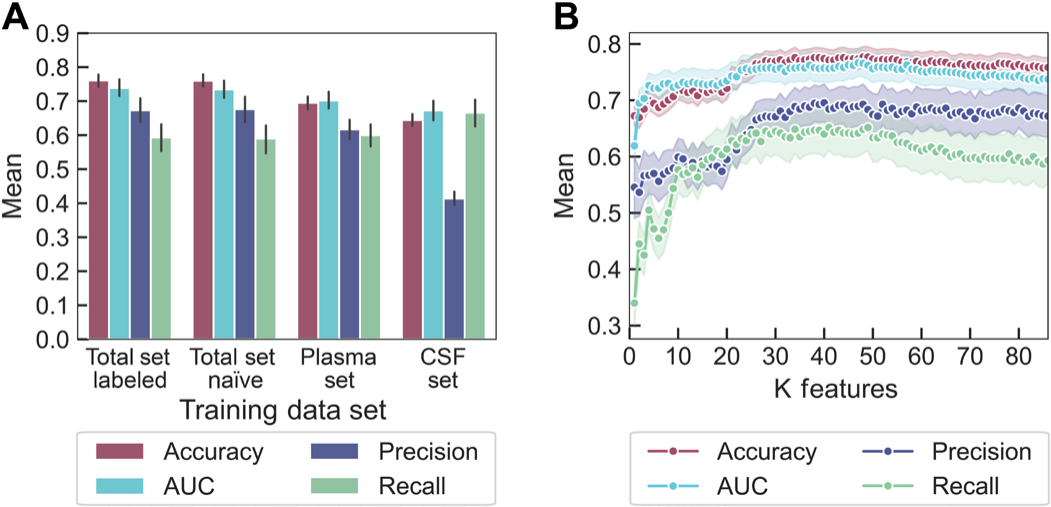
Classifier performance results on different biofluid training datasets and with varied protein feature inputs. (**A**) RFC trained on the full protein set (with or without the label of biofluid origin) or each individual biofluid (plasma or CSF). Negligible differences arise between the RFC’s ability to classify the total set with or without the biofluid label (total set labeled compared to total set naïve). Training the RFC on one biofluid and testing against the second biofluid produces similar metrics except for precision, attributable to a few proteins labeled in the corona of one biofluid but not the other. Error bars represent 95% confidence intervals. (**B**) RFC trained on the total naïve protein corona set, with features sorted by ANOVA and added to the classifier from highest to lowest importance. At approximately 40 features, classification ability begins to plateau for all metrics except recall. Shaded error bars represent 95% confidence intervals.

The first trial was with two datasets, total set labeled versus total set naïve (Fig. 2A). The only difference between these two datasets was the inclusion of one Boolean column that dictates from which biofluid a protein originated. We observe that the inclusion of this “biofluid of origin” information does not improve the classification ability on our complete dataset. Thus, we deemed this column unnecessary to include for future runs. Moreover, keeping this column would have made our classifier less generic when selecting proteins that may not be present in blood plasma or CSF.

We next trained the classifier on corona proteins present from one biofluid and attempted to predict corona proteins present from the other biofluid. For this case, instead of splitting the data 90% training/10% testing, the classifier was trained on one complete dataset, and then a subset of the second dataset was used as the testing set. We repeated this approach 100 times to generate statistics for the classifier. We report that the classifier trained on the plasma dataset results in higher accuracy (plasma, 0.695; CSF, 0.644), AUC (plasma, 0.702; CSF, 0.673), and precision (plasma, 0.617; CSF, 0.414) than the classifier trained on the CSF dataset (Fig. 2A). However, the CSF-trained set results in higher recall (CSF, 0.666; plasma, 0.600). The difference in precision arises from the inclusion of a few proteins that are present in the corona formed on (GT)_15_-SWCNTs from one biofluid and are not present in the corona formed on (GT)_15_-SWCNTs from the other biofluid (e.g., serotransferrin found in the CSF corona and haptoglobin found in the plasma corona). This discrepancy occurs because our classifier has no context of which proteins are in the corona formed from which biofluid, and thus, there is no method of adjusting for proteins displaying contradictory adsorptive behavior across biofluids. However, this classification discrepancy only occurs for a few proteins (13 proteins of 38 duplicate proteins within 174 total proteins). Including the additional feature of biofluid label did not resolve this problem (Fig. 2A), indicating that more expansive biofluid features would be necessary to correct this minor classification discrepancy.

We briefly note that both the train/test classification workflow and the classifier itself are applicable to other nanoparticle-corona systems. To demonstrate this, we used quantitative MS-based protein corona datasets for a model nanoparticle, PNPs, in blood plasma and CSF (*48*). Reasonably high metrics of classifier performance were obtained when the classifier was either trained and tested on this PNP-based dataset (fig. S5A) or by applying the classifier developed for SWCNTs directly to this different nanoparticle dataset (fig. S5B).

### Feature analysis for importance with classifier predictions

During the development of our model, 91 protein features were mined as potentially important to classify these proteins as in versus out of the nanoparticle corona (table S1). Each feature was examined for the extent of contribution to the overall classification ability of the system using an analysis of variance (ANOVA) test (Fig. 2B). This process indicates that there is a minimum of approximately 10 features to result in sufficient classification ability. If we include all 91 features, we see marginal decreases in all performance metrics. Using 38 features leads to the highest classifier performance (accuracy, 0.776; AUC, 0.758; precision, 0.695; and recall, 0.647), which we use for the remainder of the work.

**Fig. 3.**
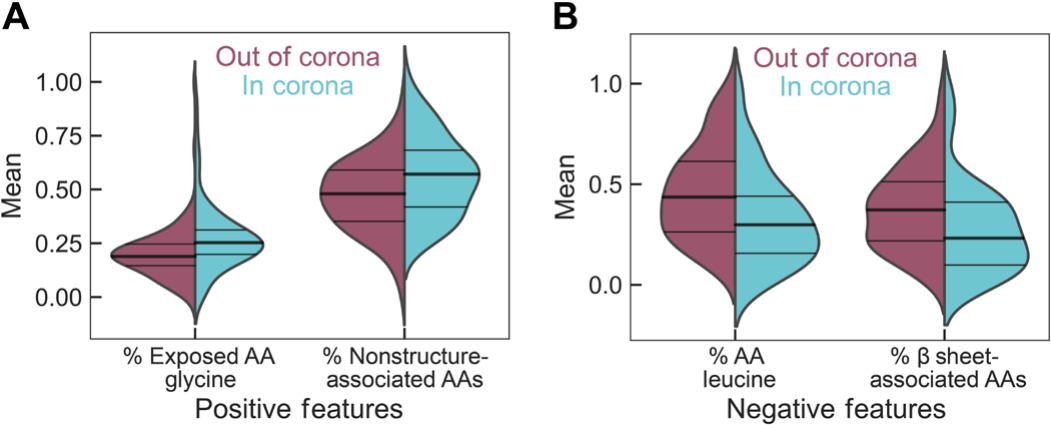
Distribution of the top four normalized feature values for proteins characterized as out of the corona phase (red) versus in the corona phase (blue) on (GT)_15_-SWCNTs. Protein features that (**A**) positively influence or (**B**) negatively influence the probability of a protein being classified as in or out of the corona are denoted by distribution shifts toward 1 or 0, respectively. (A) Positive features include (left) the fraction of solvent-exposed amino acid (AA), glycine, relative to only the solvent-exposed amino acids and (right) the fraction of amino acids not associated with any specific secondary structure motifs. (B) Negative features include (left) the fraction of amino acid, leucine, and (right) the fraction of amino acids associated with a β-sheet secondary structure.

Using the feature ranking by ANOVA, the top 10 protein features influencing protein adsorption to (GT)_15_-SWCNTs were identified (Table 1). Because RFCs do not provide correlational information (i.e., whether a high importance feature positively or negatively influences protein adsorption), we calculated basic kernel density estimates on distributions of these features and we examined how these distributions changed to hypothesize correlations (Fig. 3; top 10 feature distributions in fig. S6). As expected, these top features as ranked by ANOVA were overall in agreement with ranking by feature importance using the RFC’s ability to score individual features when constructing the classifier (table S2). We find that the fraction of solvent-exposed amino acid glycine (normalized to either the total exposed amino acid count or the total amino acid count), the fraction of amino acid glycine, and the fraction of predicted nonsecondary structure–associated amino acids correlate positively with the protein being in the corona. Conversely, the fraction of amino acid leucine and the fraction of β-sheet secondary structure–associated amino acids correlate negatively with being in the corona. The implications of these findings are explored in Discussion.

**Table 1.** Ordered importance of protein features ranked by ANOVA.

**Ranking**	**Feature**
1	% Amino acid—leucine
2	% Exposed relative to total exposed amino acids—glycine
3	% Secondary structure–associated amino acids— nonstructure associated
4	% Exposed relative to total amino acids—glycine
5	% Amino acid—glycine
6	% Secondary structure–associated amino acids—sheet
7	GRAVY score
8	% Exposed relative to total amino acids—tryptophan
9	% Exposed relative to total amino acids—histidine
10	% Exposed relative to total exposed amino acids—alanine

### Experimental validation of protein binding to SWCNTs

To assess the predictive value of our supervised learning model, we applied our classifier to rank a test set of proteins and next experimentally tested the expected protein binding order. The classifier was used to predict interaction affinity of more than 2000 total proteins [available for batch download through the UniProt database (*49*)] with (GT)_15_-SWCNT nanoparticles. These proteins represent a broad class of functions and subcellular locations and are distinct from those present in the plasma and CSF training datasets. Protein binding propensity was determined with associated binding probabilities, as summarized in table S3. We then implemented a corona exchange assay to measure real-time, in-solution protein binding dynamics on the nanotube surface, as described previously (*28*). Briefly, the ssDNA originally adsorbed on the SWCNT surface is fluorescently labeled with a Cy5 fluorophore. When near the SWCNT surface, the fluorophore is in a quenched state. Upon addition, proteins differentially bind to the SWCNT and cause various degrees of ssDNA desorption, as denoted by dequenching of the Cy5 fluorophore. Thus, fluorescence tracking of the Cy5-ssDNA provides a proxy for protein binding on the SWCNT without requiring fluorescent labeling or other modification of the protein.

**Fig. 4.**
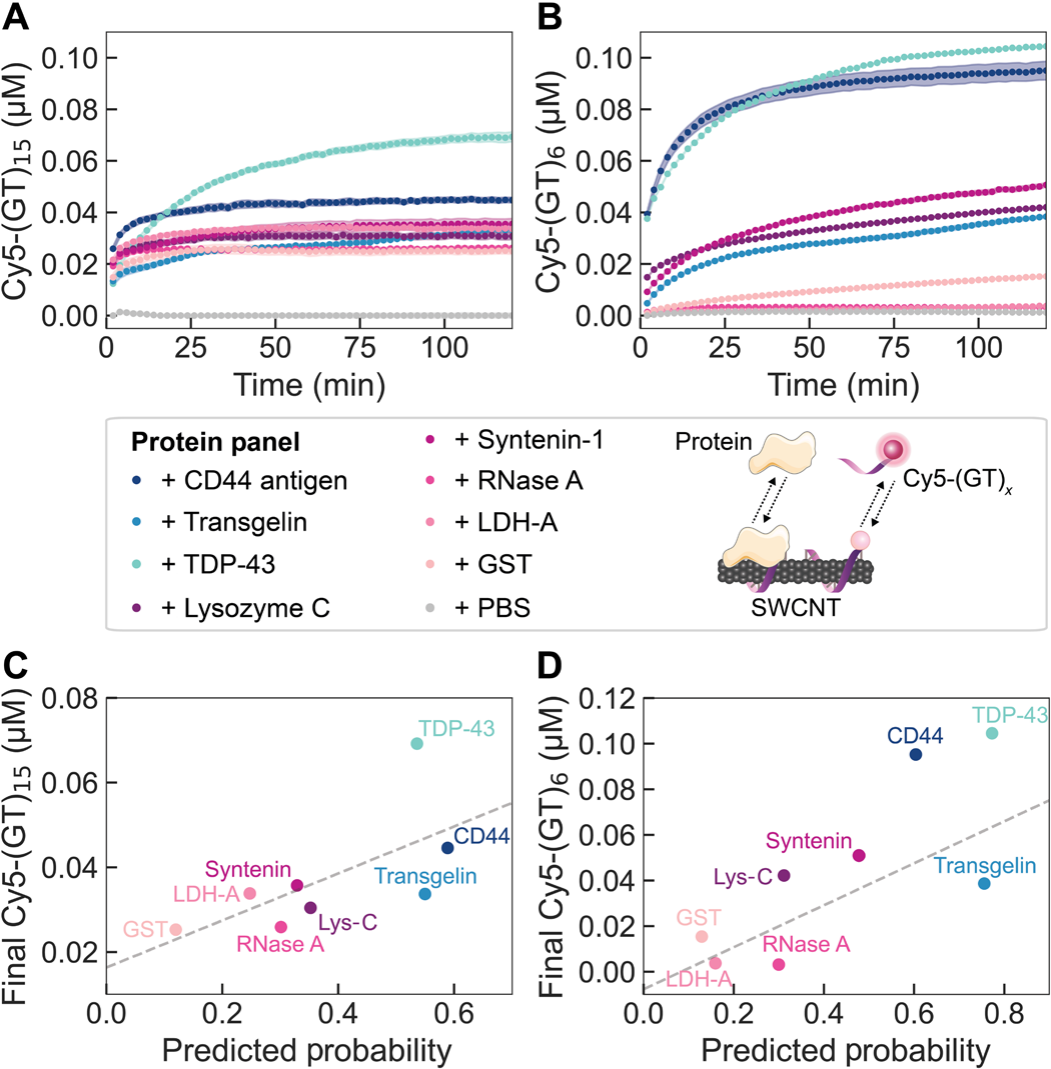
Protein corona dynamics for binding of predicted proteins to (GT)*_x_*-SWCNTs. (**A** and **B**) A corona exchange assay determines binding of a protein panel (each at 80 mg liter^−1^ final concentration) to (A) (GT)_15_-SWCNTs or (B) (GT)_6_-SWCNTs (each at 5 mg liter^−1^ final concentration). ssDNA desorption from the SWCNT serves as a proxy for protein adsorption. Proteins are predicted by the RFC to be in the corona (probability > 0.5; blue-green colors) or out of the corona (probability < 0.5; purple-pink colors). The protein panel includes CD44 antigen, transgelin, and TAR DNA binding protein 43 (TDP-43) (predicted to be in the corona) and lysozyme C (Lys-C), syntenin-1, pancreatic ribonuclease A (RNase A), l-lactate dehydrogenase A chain (LDH-A), and glutathione *S*-transferase (GST) (predicted to be out of the corona). Phosphate-buffered saline (PBS) is injected as a control, and desorbed ssDNA is normalized to this initial value. Shaded error bars represent standard error between experimental replicates (*N* = 3). (**C** and **D**) End-state–desorbed ssDNA is compared to the RFC-predicted in-corona probability for (C) (GT)_15_-SWCNTs and (D) (GT)_6_-SWCNTs.

The corona exchange assay was used to test a panel of proteins predicted to be in the corona (probability ≥ 0.5) versus out of the corona (probability < 0.5). Specifically, we tested the protein panel: CD44 antigen, transgelin, and TAR DNA binding protein 43 (TDP-43) that were predicted to adsorb to (GT)_15_-SWCNTs versus lysozyme C, syntenin-1, pancreatic ribonuclease A (RNase A), l-lactate dehydrogenase A chain (LDH-A), and glutathione *S*-transferase (GST) that were predicted to not adsorb to (GT)_15_-SWCNTs (classifier results listed in table S3). Protein adsorption based on the end-state fluorescence values predominantly matched classifier predicted outcomes of in versus out of the corona: Addition of CD44 antigen and TDP-43 both resulted in sizeable ssDNA desorption from the SWCNT surface, whereas all proteins predicted to be out of the corona produced less ssDNA desorption (Fig. 4A). Transgelin was predicted to be in the corona phase yet caused a low amount of ssDNA desorption and therefore was concluded to undergo low levels of SWCNT binding. Deviations from exact orderings of predicted outcomes arise within both groups of proteins. For example, the relative ordering of CD44 antigen as the top binding protein followed by TDP-43 is reversed. Yet, the predicted in-corona probabilities of these two proteins differ by only 4%. To provide a metric of predicted versus measured monotonicity, the Spearman’s rank-order correlation coefficient was calculated to be 0.619 (Fig. 4C), in comparison with a theoretical maximum of 0.750 for a previous protein panel comparing DNA desorption end state versus proteomic MS-derived end state (*48*). Predicted protein binding probabilities were also compared to rate constants fit to the ssDNA desorption dynamics from the SWCNT surface for each injected protein (kinetic model and fits in fig. S7). We find that there is a poor correlation between the RFC-predicted end state of protein binding and experimental dynamics of protein-SWCNT interactions. This result may be reconciled with the fact that the RFC was trained on the end-state protein corona rather than the corona composition at earlier time points and implies that the corona kinetics are influenced by distinct factors than the corona end state.

Experimental validation was repeated for the protein panel with Cy5-(GT)_6_-SWCNTs, as this shorter ssDNA oligomer is displaced more readily from the SWCNT surface and thus displays a greater spread in desorption dynamics between protein species (Fig. 4B) (*28*). Moreover, our previous study revealed that the protein corona composition formed on (GT)_6_-SWCNTs was highly similar to that on (GT)_15_-SWCNTs (*48*). To make these predictions, we trained the classifier on the protein corona datasets for (GT)_6_-SWCNTs in plasma and (GT)_15_-SWCNTs in CSF [the equivalent study of (GT)_6_-SWCNTs in CSF has not been performed because of the limited availability of CSF]. The resultant protein panel binding order was largely the same as that of Cy5-(GT)_15_-SWCNTs, with a slightly higher Spearman’s correlation coefficient of 0.762 (Fig. 4D). These results confirm that the protein binding observed experimentally is mainly driven by the protein interacting directly with the SWCNT nanoparticle surface; the shorter (GT)_6_ ssDNA merely desorbs to a greater extent and thus yields more available SWCNT surface area for protein attachment. This end-state agreement between (GT)_6_- and (GT)_15_-SWCNT datasets further accounts for any mechanistic binding differences in whether protein adsorption causes full or partial ssDNA displacement from the SWCNT surface, where the latter case may occur for longer ssDNA strands (*54*). As expected, comparison of fit rate constants versus predicted in-corona probabilities for (GT)_6_ reveals a better correlation than that of (GT)_15_, with the exception of RNase A (fig. S7).

## DISCUSSION

The classifier developed in this work provides insight into drivers of protein adsorption on SWCNTs. Our analysis of the top protein features promoting corona binding indicates that more flexible proteins are favorable to bind to (GT)_15_-SWCNTs, as inferred by high glycine content and less strict secondary structural domains. This result is in agreement with previous experimental work demonstrating that peptides and small-molecule ligands with more conformational flexibility bind more readily to carbon nanotubes (*55*, *56*). Increased adsorption propensity suggests that more flexible proteins can maximize favorable surface contacts with the highly curved SWCNT, in comparison to rigid proteins with energetic penalties associated with adopting different surface-adsorbed conformations. In the context of classic colloidal forces, these protein features may allow for maximizing favorable dispersion forces (such as van der Waals interactions) as a function of increased contact area. Flexibility itself appears in the bottom quartile of most important protein features for protein corona formation. This measure of flexibility was calculated by Vihinen *et al.* using normalized, empirically determined B factors (i.e., Debye-Waller factors) for each residue. B factors incorporate the dependence on neighboring amino acids with a nine-residue sliding window averaging approach (*57*). With this method, glycine is only the top eighth most flexible residue, posited to be because glycine frequently appears on the protein surface and interior, as well as in tight turns. The restricted mobility of glycine in the interior and turn motifs reduces the overall flexibility value. Hence, our result that high glycine content specifically located on the protein surface is an enriched feature in the corona phase indicates that protein flexibility leads to higher protein corona binding on SWCNTs. In comparison to previous literature, glycine has been found to display a relatively low magnitude, yet still favorable, free energy change upon binding to pristine SWCNTs, as determined by enhanced sampling molecular dynamics (*58*). Of note, this study was done at the scale of single amino acid analogs. Accordingly, this study disregards the full-protein structural context of each amino acid. Last, intrinsically disordered proteins have been demonstrated to disperse SWCNTs stably in the aqueous phase even under mild sonication conditions (*59*). Although the nonstructure-associated amino acid content that we report is not equivalent to intrinsically disordered domains, our result is in line with these previous experimental findings and further supports the role of protein flexibility in corona binding.

In contrast, our analysis of top protein features that deter corona binding reveals that proteins high in the aliphatic, hydrophobic amino acid leucine and proteins with more planar β-sheet character are not expected to bind to (GT)_15_-SWCNTs. For physical context, (GT)_15_ ssDNA is observed to wrap helically around SWCNTs based on both experiment (*60*, *61*) and modeling (*62*, *63*), although only covering ~2 to 25% of the aromatic SWCNT surface (*62*, *64*, *65*). Accordingly, the finding that hydrophobic leucine does not increase SWCNT binding is not intuitive, considering that the SWCNT surface is highly hydrophobic. Yet, this result recapitulates previous literature that nonspecific hydrophobic interactions alone do not drive corona binding (*55*, *58*, *66*, *67*); rather, aromatic hydrophobic amino acids, especially tryptophan, are repeatedly found to be the highest binders to SWCNTs (*55*, *66*–*69*). The RFC highlighted the fraction of exposed tryptophan as the fifth most favorable feature for corona binding, although total (both exposed and buried) aromatic amino acid contents (tryptophan, tyrosine, or phenylalanine) were not ranked as top features. In studies of isolated amino acids or short peptide sequences, aromatic amino acids seemingly drive adsorption to SWCNTs via π-π interactions with the SWCNT surface. However, in the full protein context, these aromatic amino acids may not be sufficient to initiate protein contact with the SWCNT surface, as these hydrophobic amino acids are expected to be predominantly buried in the folded protein core. This result of our analysis is important to consider in extrapolating the conclusions drawn from single- or few-peptide adsorption experiments to the expected outcomes of whole-protein binding: These key residues, in this case, tryptophan, should be located on the protein surface to promote corona binding.

The reasoning for the highly ranked, inverse relationship between leucine-rich proteins and SWCNT binding may be that leucine content is a proxy for hydrophobic core stability, such as in α-helix motifs, and that more internally stable proteins are less likely to reorient and bind to nearby nanoparticles. This analysis is further supported by high grand average hydropathy (GRAVY) score (i.e., net protein hydrophobicity) appearing as the third most important feature for proteins that do not enter the corona phase. Also in line with this protein reorientation argument, the finding that high content of amino acids associated with β-sheet structures leads to low protein adsorption indicates the difficulty for planar protein secondary structures to adapt to the highly curved SWCNT surface. For context, each SWCNT has an extremely high aspect ratio, with an average diameter of 1 nm and length of 500 nm. Our result is in agreement with previous work demonstrating that the high curvature of carbon nanotubes must be aligned at the amino acid level of proteins, less the secondary structure level (*55*, *66*). Overall, the identification of these features is important in helping to predict high biofouling protein types or rationally selecting proteins to bind to nanotubes before testing them experimentally.

Previously, we linearly regressed the log-fold change (ratio of protein amount in the corona versus in the native biofluid) against physicochemical protein properties to understand protein features that govern corona formation (*48*). In this analysis, high leucine content was similarly determined to be less favorable for protein adsorption to (GT)_15_-SWCNTs. High glycine content was found to be associated with more favorable protein adsorption when included in the regression analysis. However, glycine contribution was not evaluated in the original regression because of correlation with other protein features, as the calculated variance inflation factor was greater than the set threshold value (*48*). Hence, glycine content impact could not be deconvoluted from other protein properties. This analysis highlights a benefit of the current RFC over the previously applied linear regression approach, where co-dependent variables must be proactively excluded in the latter case. It should further be noted that secondary structure features were not included in the protein property database for the linear regression analysis because of data sparsity, whereas here we implement BioPython to predict such features from the amino acid sequence without relying on protein structure availability.

The corona exchange assay enabled us to quickly test our classifier against a panel of potential protein binders. Examining the protein identities, note that lysozyme has previously been demonstrated to strongly interact with and disperse pristine carbon nanotubes, in which hydrophobic aromatic amino acids (tryptophan and tyrosine) and cationic amino acids (arginine and lysine) are hypothesized to drive adsorption (*70*–*74*). Yet, here, we find that lysozyme interacts less with predispersed ssDNA-SWCNTs based on the corona exchange results. Therefore, strong lysozyme-SWCNT interaction may hinge upon energetic input used during the initial SWCNT dispersion process, which likely denatures lysozyme to expose more aromatic residues. This result is important in suggesting that some proteins can only be adsorbed to SWCNT nanoparticles in a partially or fully denatured state, likely compromising their enzymatic activities or protein functions. Another protein of note is CD44, which is overexpressed on the surface of cancer-initiating cells (*75*). Toward our goal of facilitating nano-bio construct design, the innate affinity of the SWCNT for CD44 could be applied to construct a cell-targeted nanotube delivery system.

In sum, we applied supervised learning methods and developed a classifier to predict protein adsorption on ssDNA-functionalized SWCNTs with 78% accuracy, 76% AUC, 70% precision, and 65% recall. Ensemble methods performed better in the corona classification task, and an RFC scheme was ultimately chosen and optimized. We expand upon prior predictive protein corona work by (i) leveraging quantitative protein corona data (*48*), (ii) redefining corona thresholding, with corresponding prediction probabilities, (iii) establishing a method for classifying proteins based solely on the amino acid sequence of the protein, and (iv) experimentally confirming adsorption with unmodified proteins in the solution phase (*28*). We find that no single or small group of protein physicochemical features best determines placement in the corona. Rather, nearly 40 features are useful for protein classification when optimizing all four metrics of accuracy, AUC, precision, and recall. We confirm the need for these protein features by staging them into the classifier feature-by-feature and revalidating our model. Using kernel density estimates, we elucidate protein feature correlation with proteins binding or not binding to SWCNTs. We find that proteins with high solvent-exposed glycine content and more nonstructure-associated amino acid content (serving as proxies for protein flexibility) bind in the SWCNT corona, while proteins with high leucine content and β sheet–associated amino acid content (serving as proxies for internal protein stability) do not. The classifier then enabled rapid determination of proteins predicted to enter the corona phase from a new protein set, as validated experimentally with a corona exchange assay. Our machine learning algorithm allows us to quickly parse protein properties from a publicly available database to determine protein features and proteins of interest for corona formation on SWCNTs.

We intend for this work to support the development of predictive protein corona models that will inform heuristics to rationally select proteins for nanoparticle complexation or to predict biofouling of nanotechnologies. We demonstrate that the workflow and the developed classifier itself can be translatable to different nanoparticles, such as PNPs. Our model uses amino acid sequence–based prediction of protein corona formation, which could be generalizable across a wide range of bioenvironments. Recent advances in prediction of protein properties from protein sequences alone are promising toward refinement of the protein database we have curated for this classifier, enabling inclusion of biological protein properties that are not reliant on experimental study and manual sequence annotation (*76*). Model accuracy could accordingly be improved by adding structural and geometric protein parameters, such as better-predicted structural motifs, local protein surface curvature, and surface patch hydrophobicity. In the extension of this work, nanoparticle features may be included to enable classification on more nanoparticle types. Such nanoparticle features should be readily accessible to retain the triviality of classifying future systems. Ultimately, in silico protein corona prediction will support the design of nanotechnologies that can be more seamlessly implemented in biological systems with reduced need for experimental MS-based proteomic characterization and analysis. The ability to predict adsorption of specific proteins will enable connection to downstream cellular responses, toxicity outcomes, and overall nanotechnology functionality. The developed classifier provides a preliminary tool for both predicting key proteins expected to take part in biofouling and rapid prescreening of protein candidates in rationally designed nanobiotechnologies.

## MATERIALS AND METHODS

### Database development

Protein information was downloaded from UniProt (*49*), including amino acid sequences (FASTA format) and sequence annotations. Amino acid sequences were used to generate a series of physicochemical protein properties using BioPython’s Protein Analysis module (table S1) (*50*). Amino acid sequences were additionally analyzed by NetSurfP 2.0 (*51*) to determine solvent accessibility, including relative solvent accessibility (RSA), absolute solvent accessibility (ASA), and fractions of each amino acid exposed surface area relative to either all amino acids or only other exposed amino acid surface area. To collate these data, we programmatically created submissions from UniProt protein sequence entries to NetSurfP 2.0, aligning with our goal of creating an easily expandable database. The resulting data were processed and merged with the BioPython analysis. The complete database was scaled with the MinMaxScalar from Scikit-Learn (*77*) before being subset and fit to the classification model. Code for this and all subsequent sections can be found in the GitHub link provided and in (*78*).

### Criteria for in-corona placement

Using the method described previously for protein corona studies by LC-MS/MS (*48*), quantitative data were obtained for proteins adsorbing to (GT)_15_-SWCNTs in two different human biofluids: blood plasma and CSF. First, proteins with abundances (*A*_corona_) greater than the control of protein abundances in biofluids alone (*A*_biofluid_) were assigned as in the corona (i.e., enriched in the corona relative to the biofluid). Second, an exponential decay, *n* = *n*_0_ exp(− *kA*), was fit to the distribution of abundances for the remaining proteins, where *n*_0_ and *k* are fitting parameters. An abundance threshold (*A*_threshold_) was selected at a value where the exponential decay fell to a value of *n*_0_ exp(− *p*), or *A*_threshold_ = *p*/*k*, where *p* is an optimization parameter. Proteins with an abundance greater than *A*_threshold_ were assigned as being in the corona. We varied *p* between 0 and 3.5 and chose the value 2.25, which optimized the performance of the classifier following training (Fig. 1B) and was used for the remainder of the analysis. Corona thresholding was originally completed with Otsu’s method, a technique generally implemented for image thresholding (*79*). However, using Otsu’s method resulted in only three to five proteins placed in the corona for each biofluid. Although the classifier was highly accurate at identifying these proteins, the number of proteins selected was not fully representative of the corona and we accordingly implemented our modified thresholding method described above.

### Classifier selection

The use of an RFC, logistic regression, bagging classifier, gradient boosting classifier, AdaBoost classifier, and XGBoost classifier was evaluated. The RFC, logistic regression, bagging classifier, gradient boosting classifier, and AdaBoost classifier were imported from Scikit-Learn (*77*). The XGBoost classifier was imported from XGBoost (*80*) for use with Scikit-Learn. AdaBoost and bagging classifiers were tested with an underlying support vector machine, decision tree, and logistic regression. The gradient boosting classifier was tested with an underlying decision tree. XGBoost was tested with an underlying decision tree and 100 parallel trees.

The RFC performed best and was accordingly chosen for the remainder of the work. The classifier (with 700 trees and an entropy criterion) was next validated using a stratified shuffle split (100 repeats) validation to ensure consistent levels of the minority class. The minority class here is the in-corona class, which has fewer proteins than the out-of-corona class. The shuffle split retained 10% of the dataset for corona validation. The training split was augmented with entries developed from SMOTE (minority/majority ratio of 0.7:1 with 12 k-neighbors), as detailed in the main text. Results were collected for each fold. For cross-biofluid tests, the percentage of proteins in the test set was varied to keep the same number of proteins in the test set equal to 10% of the total number of proteins used for mixed biofluid cases. The adjusted value was set by scaling 10% by a factor of the total number of proteins divided by the number of proteins in the test biofluid (plasma, 1.55; CSF, 2.81).

### Hyperparameter tuning

Using Scikit-Learn’s GridSearchCV (*77*), a wide range of hyperparameters, such as number or depth of trees, were tested with the classifier. With each set of hyperparameters, the model was validated using the method dictated in the previous section and scored. The classifier was chosen with the hyperparameters optimized for precision using GridSearchCV. A full list of hyperparameters can be found at the GitHub link provided and in (*78*).

### Dimensionality reduction

To understand the effects of each feature (i.e., variable describing the protein) on the total system, features were ranked using Scikit-Learn’s SelectKBest function (*77*). Using the ranking established from SelectKBest, the database features were unmasked one-by-one running the classifier as described in the “Classifier selection” section until all features had been added in. Metric results were saved, and statistics were calculated.

### Prediction targets

The classifier was tested against a list of 996 cytoplasmic proteins and 999 nuclear proteins [available for batch download through the UniProt database (*49*)], together with 45 readily accessible proteins or proteins of interest for SWCNT-based sensing and delivery applications. Amino acid sequences for these proteins were downloaded from UniProt and processed through the database development workflow described above. This complete protein database was then processed through the classifier *k* + 1 times. The first *k* times were completed through the described *k*-fold validation using the combined datasets for (GT)_15_-SWCNTs in plasma and CSF as the training and verification data. Predictions were recorded at the end of each fold. When protein targets were run, all available data were used to train the classifier. This last classifier then provided predictions on the test proteins, as listed in table S3.

### Synthesis of ssDNA-SWCNTs

Suspensions of SWCNTs with fluorophore-labeled ssDNA [Cy5-(GT)_15_ or Cy5-(GT)_6_] were prepared with 0.2 mg of mixed-chirality SWCNTs (small-diameter HiPco SWCNTs, NanoIntegris) and 20 μM ssDNA (3′ Cy5-labeled custom ssDNA oligos with high-performance liquid chromatography purification, Integrated DNA Technologies Inc.; excitation, 648 nm; emission, 668 nm) added in 1 ml total volume of 0.1× phosphate-buffered saline (PBS; note that 1× is 137 mM NaCl, 2.7 mM KCl, 10 mM Na_2_HPO_4_, and 1.8 mM KH_2_PO_4_) (*28*). This mixture was probe tip–sonicated for 10 min in an ice bath (3 mm probe tip at 50% amplitude, 5 to 6 W, Cole-Parmer Ultrasonic Processor). Cy5-ssDNA-SWCNT suspensions were centrifuged to pellet insoluble SWCNT bundles and contaminants [16,100 relative centrifugal force (rcf), 30 min]. The supernatant-containing product was collected, and Cy5-ssDNA-SWCNT concentration was calculated with measured sample absorbance at 910 nm (NanoDrop One, Thermo Fisher Scientific) and the empirical extinction coefficient, ε_910nm_ = 0.02554 liter mg^−1^ cm^−1^ (*81*). Cy5-ssDNA-SWCNTs were stored at 4°C until use, at which point the solution was diluted to a working concentration of 10 mg liter^−1^ in 1× PBS ≥2 hours before use.

### Preparation of proteins

Proteins were sourced as listed in Table 2. Lyophilized proteins were reconstituted to the listed concentration in PBS, tilting intermittently to dissolve for 15 min, and filtering with 0.2-μm syringe filters (cellulose acetate membrane, VWR International). All proteins were purified with desalting columns (Zeba Spin Desalting Columns, 0.5 ml with 7-kDa molecular weight cutoff; Thermo Fisher Scientific) by washing with PBS three times (centrifuging 1500 rcf, 1 min), centrifuging with sample (1500 rcf, 2 min), and retaining sample in flow-through solution. Resulting protein concentration was measured with the Qubit Protein Assay (Thermo Fisher Scientific).

**Table 2.** Purchased protein specifications.

**Protein**	**Manufacturer**	**Catalog no.**	**Lot no.**	**Source**	**Notes**
CD44 antigen	Acro Biosystems	CD4-H5226	616-784F1-G8	Human, expressed in HEK293	6× His tag; >95% purity
Transgelin	MyBioSource	MBS144070	1011PTAGLN30	Recombinant human, expressed in *Escherichia coli*	20× His tag; >85% purity
TDP-43	R&D Systems	AP-190	22675420A	Recombinant human, expressed in *E. coli*	>85% purity
Lysozyme C	Sigma-Aldrich	L2879	SLCF2361	From chicken egg white	≥80% purity
Syntenin-1	Novus Biologicals	NBP1-50893	1082301	Recombinant human, expressed in *E. coli*	6× His tag; >90% purity
RNase A	New England Biolabs	T3018L		Purified from cow pancreas	
LDH-A	Sigma-Aldrich	10127230001	42032824	From rabbit muscle	
GST	Abcam	ab86775	GR3377596-1	Recombinant mouse, expressed in *E. coli*	>95% purity

### Corona exchange assay

Corona dynamics were measured as described previously (*28*). Briefly, equal volumes (25 μl) of ssDNA-Cy5-SWCNT and FAM-protein at 2× working concentration were added via multichannel pipette into a 96-well polymerase chain reaction (PCR) plate (Bio-Rad) and mixed by pipetting. The PCR plate was sealed with an optically transparent adhesive seal (Bio-Rad) and briefly spun down on a benchtop centrifuge. Fluorescence was measured as a function of time using a Bio-Rad CFX96 real-time quantitative PCR system, scanning all manufacturer set color channels (FAM, HEX, Texas Red, Cy5, Quasar 705) every 30 s at 22.5°C, with lid heating off. Fluorescence time series were analyzed without default background correction. Fluorophore dequenching indicates that the 3′ end of the Cy5-tagged ssDNA was displaced from the SWCNT surface and may not indicate complete ssDNA strand desorption.

## Supplementary Materials

### This PDF file includes:

Figs. S1 to S7 Tables S1 to S3 Legend for data S1 References
