# Supplementary material for: Supervised learning model predicts protein adsorption to carbon nanotubes
Source: Sci Adv. 2022 Jan 7;8(1):eabm0898. doi: 10.1126/sciadv.abm0898 (PMC8741178; doi:10.1126/sciadv.abm0898)
Supplement: Supplementary file 1 — Figs. S1 to S7 Tables S1 to S3 Legend for data S1 References [file sciadv.abm0898_sm.pdf]

Science Advances
AAAS

Supplementary Materials for

Supervised learning model predicts protein adsorption to carbon nanotubes

Nicholas Ouassil, Rebecca L. Pinals, Jackson Travis Del Bonis-O'Donnell,
Jeffrey W. Wang, Markita P. Landry*

*Corresponding author. Email: landry@berkeley.edu

Published 7 January 2022, *Sci. Adv.* **8**, eabm0898 (2022)
DOI: 10.1126/sciadv.abm0898

The PDF file includes:

Figs. S1 to S7
Tables S1 to S3
Legend for data S1
References

Other Supplementary Material for this manuscript includes the following:

Data S1

Table S1. Protein property list.

Protein Property	Calculated By	Implementation in Code
% Amino Acid - Alanine (A)	BioPython	frac_aa_A
% Amino Acid - Cysteine (C)	BioPython	frac_aa_C
% Amino Acid - Aspartic Acid (D)	BioPython	frac_aa_D
% Amino Acid - Glutamic Acid (E)	BioPython	frac_aa_E
% Amino Acid - Phenylalanine (F)	BioPython	frac_aa_F
% Amino Acid - Glycine (G)	BioPython	frac_aa_G
% Amino Acid - Histidine (H)	BioPython	frac_aa_H
% Amino Acid - Isoleucine (I)	BioPython	frac_aa_I
% Amino Acid - Lysine (K)	BioPython	frac_aa_K
% Amino Acid - Leucine (L)	BioPython	frac_aa_L
% Amino Acid - Methionine (M)	BioPython	frac_aa_M
% Amino Acid - Asparagine (N)	BioPython	frac_aa_N
% Amino Acid - Proline (P)	BioPython	frac_aa_P
% Amino Acid - Glutamine (Q)	BioPython	frac_aa_Q
% Amino Acid - Arginine (R)	BioPython	frac_aa_R
% Amino Acid - Serine (S)	BioPython	frac_aa_S
% Amino Acid - Threonine (T)	BioPython	frac_aa_T
% Amino Acid - Valine (V)	BioPython	frac_aa_V
% Amino Acid - Tryptophan (W)	BioPython	frac_aa_W
% Amino Acid - Tyrosine (Y)	BioPython	frac_aa_Y
GRAVY score	BioPython	gravy
Aromaticity	BioPython	aromaticity
Instability Index	BioPython	instability_index
Flexibility - Mean	BioPython	flexibility_mean
Flexibility - Standard Deviation	BioPython	flexibility_std
Flexibility - Variance	BioPython	flexibility_var
Flexibility - Max	BioPython	flexibility_max
Flexibility - Min	BioPython	flexibility_min
Flexibility - Median	BioPython	flexibility_median
Isoelectric Point	BioPython	isoelectric_point
% Secondary Structure-Associated Amino Acids – Helix (V, I, Y, F, W, L)	BioPython	secondary_structure_fraction_helix
% Secondary Structure-Associated Amino Acids – Turn (N, P, G, S)	BioPython	secondary_structure_fraction_turn
% Secondary Structure-Associated Amino Acids – Sheet (E, M, A, L)	BioPython	secondary_structure_fraction_sheet
% Secondary Structure-Associated Amino Acids - Non-Structure Associated	BioPython	secondary_structure_fraction_disordered
Length	BioPython	length
Molecular Weight	BioPython	molecular_weight
% Amino Acids Exposed	NetSurfP	fraction_exposed
% Amino Acids Buried	NetSurfP	fraction_buried
% Exposed Nonpolar Amino Acids / Total Amino Acids	NetSurfP	fraction_exposed_nonpolar_total

% Exposed Nonpolar Amino Acids / Total Exposed	NetSurfP	fraction_exposed_nonpolar_exposed
% Exposed Polar Amino Acids / Total Amino Acids	NetSurfP	fraction_exposed_polar_total
% Exposed Polar Amino Acids / Total Exposed	NetSurfP	fraction_exposed_polar_exposed
Relative Surface Area (RSA) - Mean	NetSurfP	rsa_mean
Relative Surface Area - Median	NetSurfP	rsa_median
Relative Surface Area - Standard Deviation	NetSurfP	rsa_std
Absolute Surface Area (ASA) - Sum	NetSurfP	asa_sum
% Exposed Amino Acid A / Total Amino Acids	NetSurfP	fraction_total_exposed_A
% Exposed Amino Acid C / Total Amino Acids	NetSurfP	fraction_total_exposed_C
% Exposed Amino Acid D / Total Amino Acids	NetSurfP	fraction_total_exposed_D
% Exposed Amino Acid E / Total Amino Acids	NetSurfP	fraction_total_exposed_E
% Exposed Amino Acid F / Total Amino Acids	NetSurfP	fraction_total_exposed_F
% Exposed Amino Acid G / Total Amino Acids	NetSurfP	fraction_total_exposed_G
% Exposed Amino Acid H / Total Amino Acids	NetSurfP	fraction_total_exposed_H
% Exposed Amino Acid I / Total Amino Acids	NetSurfP	fraction_total_exposed_I
% Exposed Amino Acid K / Total Amino Acids	NetSurfP	fraction_total_exposed_K
% Exposed Amino Acid L / Total Amino Acids	NetSurfP	fraction_total_exposed_L
% Exposed Amino Acid M / Total Amino Acids	NetSurfP	fraction_total_exposed_M
% Exposed Amino Acid N / Total Amino Acids	NetSurfP	fraction_total_exposed_N
% Exposed Amino Acid P / Total Amino Acids	NetSurfP	fraction_total_exposed_P
% Exposed Amino Acid Q / Total Amino Acids	NetSurfP	fraction_total_exposed_Q
% Exposed Amino Acid R / Total Amino Acids	NetSurfP	fraction_total_exposed_R
% Exposed Amino Acid S / Total Amino Acids	NetSurfP	fraction_total_exposed_S
% Exposed Amino Acid T / Total Amino Acids	NetSurfP	fraction_total_exposed_T
% Exposed Amino Acid V / Total Amino Acids	NetSurfP	fraction_total_exposed_V
% Exposed Amino Acid W / Total Amino Acids	NetSurfP	fraction_total_exposed_W
% Exposed Amino Acid Y / Total Amino Acids	NetSurfP	fraction_total_exposed_Y
% Exposed Amino Acid A / Total Exposed	NetSurfP	fraction_exposed_exposed_A
% Exposed Amino Acid C / Total Exposed	NetSurfP	fraction_exposed_exposed_C
% Exposed Amino Acid D / Total Exposed	NetSurfP	fraction_exposed_exposed_D
% Exposed Amino Acid E / Total Exposed	NetSurfP	fraction_exposed_exposed_E
% Exposed Amino Acid F / Total Exposed	NetSurfP	fraction_exposed_exposed_F
% Exposed Amino Acid G / Total Exposed	NetSurfP	fraction_exposed_exposed_G
% Exposed Amino Acid H / Total Exposed	NetSurfP	fraction_exposed_exposed_H
% Exposed Amino Acid I / Total Exposed	NetSurfP	fraction_exposed_exposed_I
% Exposed Amino Acid K / Total Exposed	NetSurfP	fraction_exposed_exposed_K
% Exposed Amino Acid L / Total Exposed	NetSurfP	fraction_exposed_exposed_L
% Exposed Amino Acid M / Total Exposed	NetSurfP	fraction_exposed_exposed_M
% Exposed Amino Acid N / Total Exposed	NetSurfP	fraction_exposed_exposed_N
% Exposed Amino Acid P / Total Exposed	NetSurfP	fraction_exposed_exposed_P
% Exposed Amino Acid Q / Total Exposed	NetSurfP	fraction_exposed_exposed_Q
% Exposed Amino Acid R / Total Exposed	NetSurfP	fraction_exposed_exposed_R
% Exposed Amino Acid S / Total Exposed	NetSurfP	fraction_exposed_exposed_S
% Exposed Amino Acid T / Total Exposed	NetSurfP	fraction_exposed_exposed_T

% Exposed Amino Acid V / Total Exposed	NetSurfP	fraction_exposed_exposed_V
% Exposed Amino Acid W / Total Exposed	NetSurfP	fraction_exposed_exposed_W
% Exposed Amino Acid Y / Total Exposed	NetSurfP	fraction_exposed_exposed_Y
Sum of Absolute Surface Area / Total Mass	NetSurfP	asa_sum_normalized
% Secondary Structure - Helix	NetSurfP	nsp_secondary_structure_helix
% Secondary Structure - Sheet	NetSurfP	nsp_secondary_structure_sheet
% Secondary Structure - Coil	NetSurfP	nsp_secondary_structure_coiled
% Secondary Structure - Disordered	NetSurfP	nsp_disordered

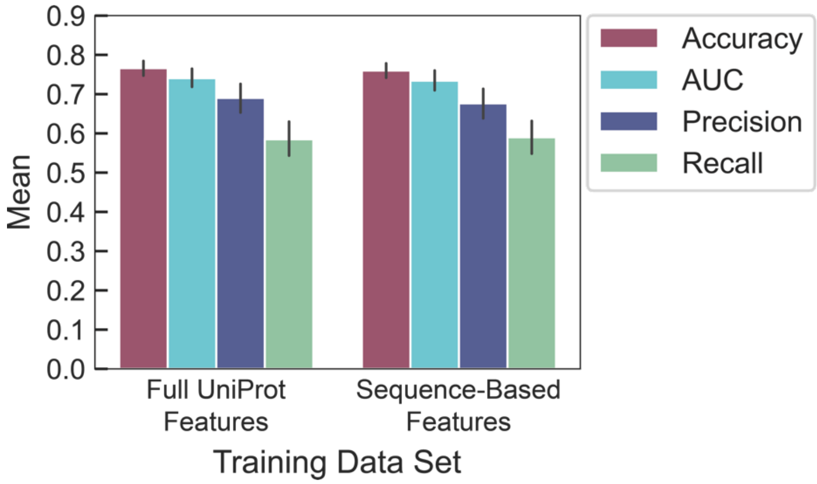

Fig. S1. Comparison of classifier performance using all biological and physicochemical protein features available through UniProt (left) vs. using only amino acid-sequence derived protein features as listed in Table S1 (right). Error bars represent 95% confidence intervals.

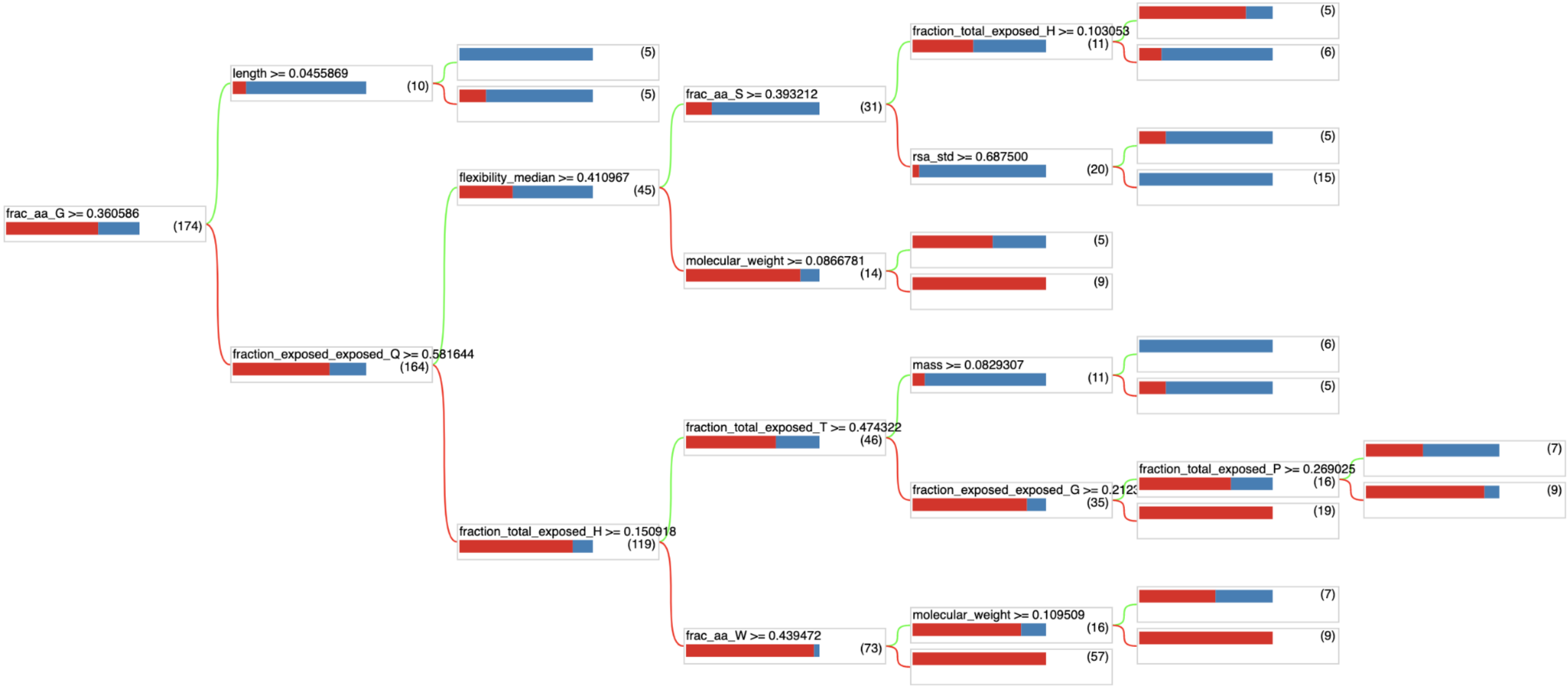

Fig. S2. Example random forest tree (1 of 300 trees) to visualize how a random forest classifier makes its decision. Each tree is traversed left to right. If the node criteria evaluates to true, the green path is taken. If the node criteria evaluates to false, the red path is taken. This process is repeated until the protein reaches a terminal node then a vote is recorded. Total votes among all trees are tallied and a decision is made by majority rules. Bars at each node indicate the percentage of data at that node that is in each phase (blue - in corona, red - out of corona). Parentheses indicate the number of data points at each node. Created using TensorFlow-Decision Forests (*82*).

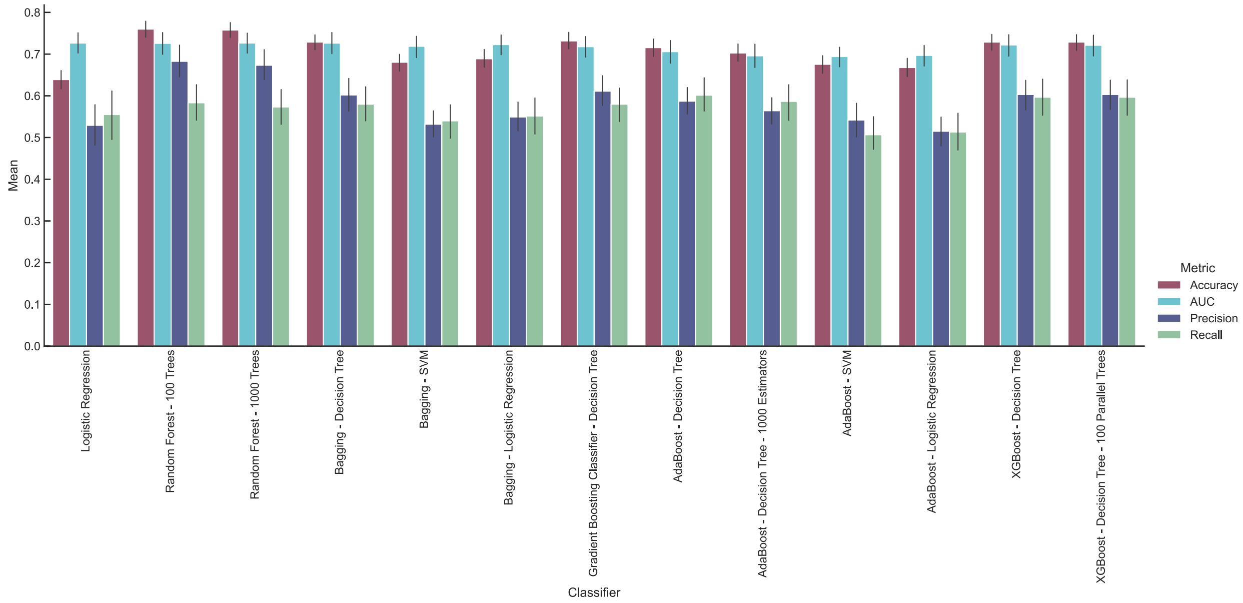

Fig. S3. Comparison of output metrics for a panel of classifiers. Classifiers tested: logistic regression, random forest (100 or 1000 trees), bagging (decision tree, SVM, logistic regression), gradient boosting classifier (decision tree), AdaBoost (decision tree and with 1000 estimators, SVM, logistic regression), and XGBoost (decision tree and with 100 parallel trees). Corresponding classifier metrics are reported for each: accuracy, area under the receiver operating curve (AUC), precision, and recall. Error bars represent 95% confidence intervals.

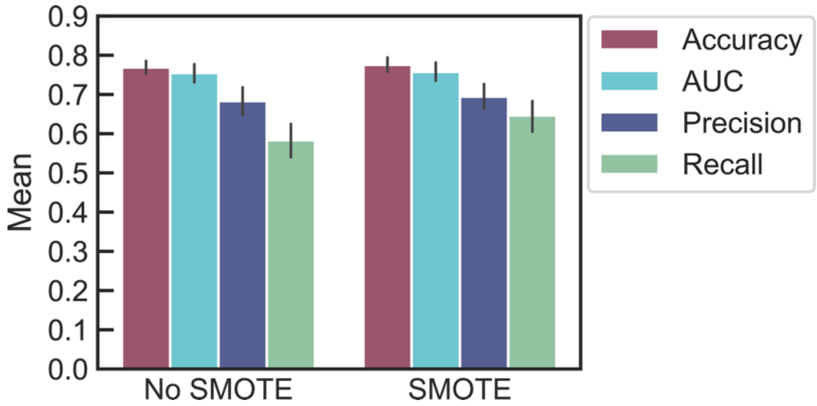

Fig. S4. Comparison of classifier performance prior to (left) and after (right) introducing a synthetic minority over-sampling technique (SMOTE). SMOTE Minority/Majority ratio is set to 0.7/1 with 12 k-neighbors for estimation. The classifier uses the top 38 protein features to achieve optimal performance. Error bars represent 95% confidence intervals.

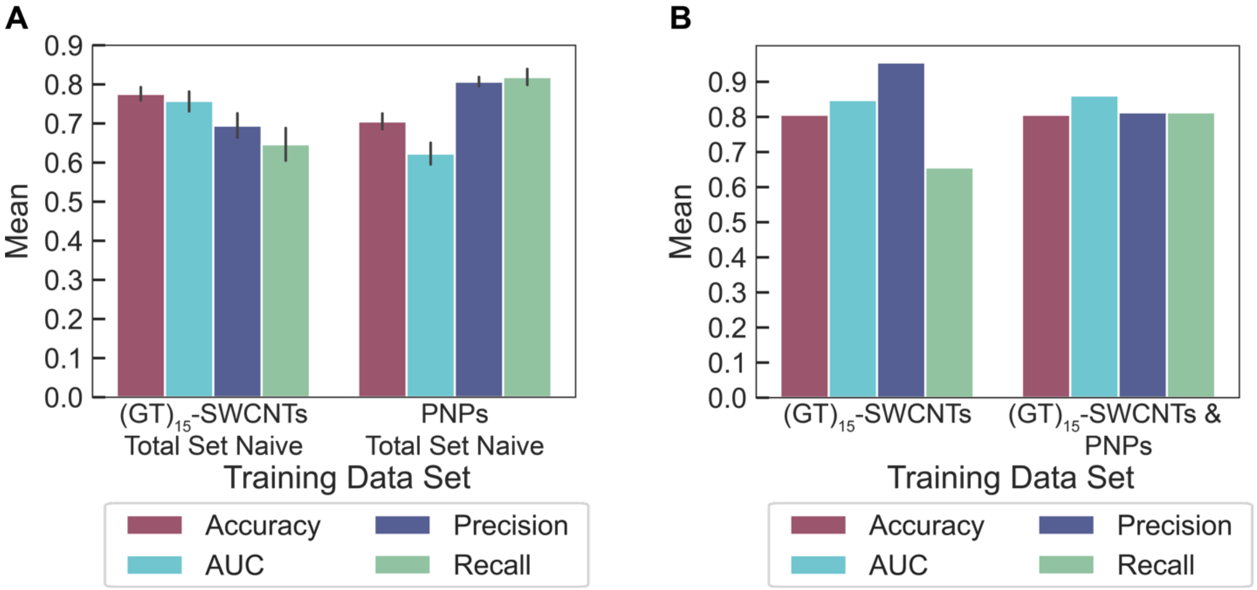

Fig. S5. Extension of classifier to model protein corona on polystyrene nanoparticles (PNPs). **(A)** Full classification workflow is applied on either the $(GT)_{15}$-SWCNT protein corona dataset (left) or PNP protein corona dataset (right), for each nanoparticle in blood plasma and CSF. **(B)** Classifier is applied to predict the protein corona formed on PNPs in CSF, with the training set as either $(GT)_{15}$-SWCNTs in blood plasma and CSF (left) or this in addition to PNPs in blood plasma (right). The classifier uses the top 38 protein features to achieve optimal performance. Error bars represent 95% confidence intervals.

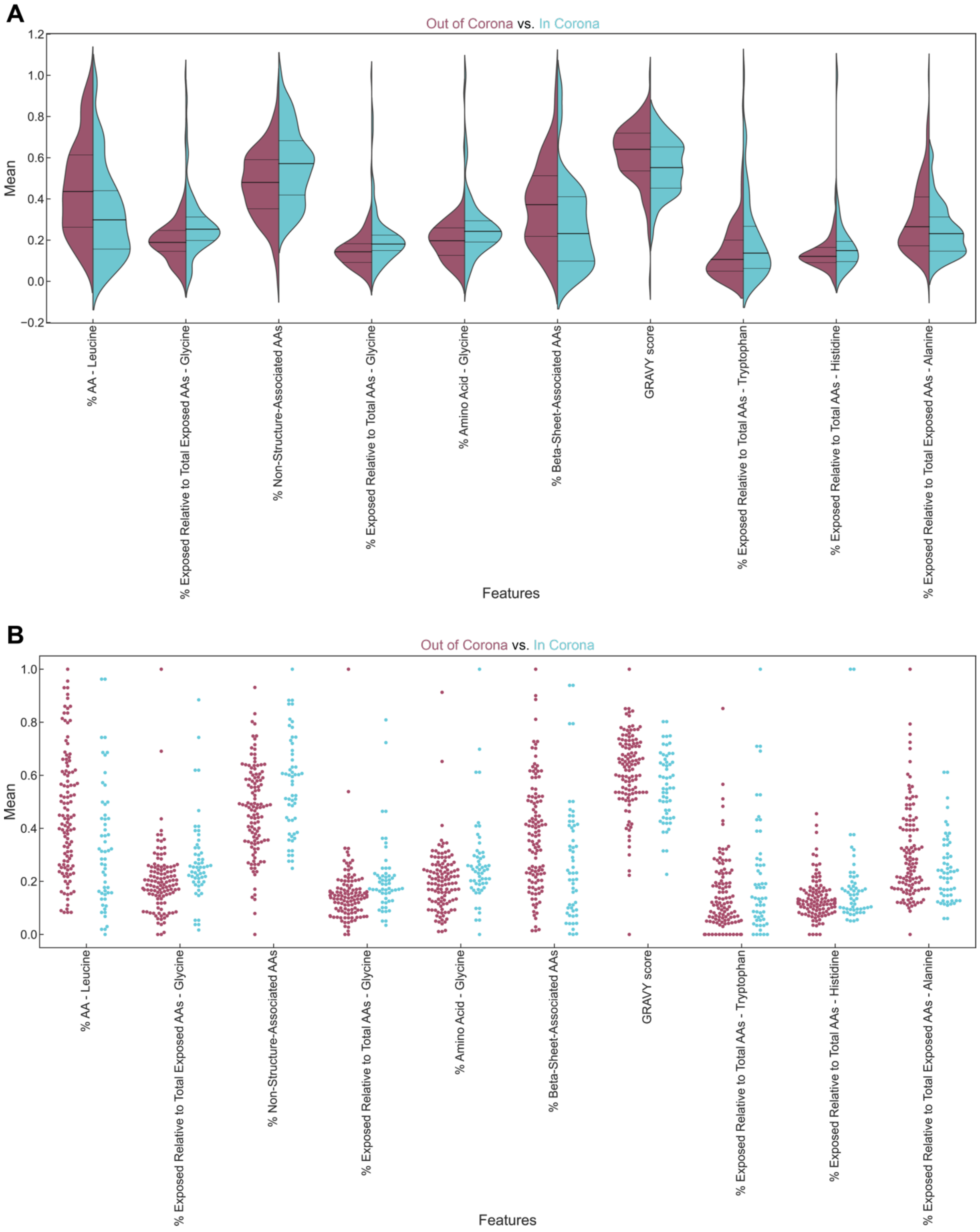

Fig. S6. Distribution of the top ten normalized feature values for proteins characterized as out of the corona phase (red) vs. in the corona phase (blue) on $(GT)_{15}$-SWCNTs. Protein features that positively influence or negatively influence the probability of a protein being classified as in the corona are denoted by distribution shifts toward 1 or 0, respectively. Protein features are

represented with **(A)** violin plots and **(B)** scatter distribution plots. GRAVY score represents the grand average of hydropathy value (*83*).

Table S2. Ordered importance of protein features by weight.

Ranking	Top Features	Weight
1	% Exposed relative to total exposed amino acids - glycine	0.03321
2	Instability index	0.02961
3	% Exposed relative to total amino acids - glycine	0.02933
4	% Amino acid - leucine	0.02773
5	GRAVY score	0.02353
6	% Secondary structure-associated amino acids - sheet	0.02158
7	% Amino acid - tryptophan	0.01734
8	% Secondary structure-associated amino acids - helix	0.01701
9	% Amino acid - glycine	0.01472
10	Aromaticity	0.01466
**Ranking**	**Bottom Features**	**Weight**
1	Length	0.00713
2	% Exposed relative to total exposed amino acids - valine	0.00705
3	% Exposed relative to total amino acids - threonine	0.00704
4	% Amino acid – aspartic acid	0.00700
5	% Amino acid – glutamic acid	0.00688
6	% Secondary structure - helix	0.00688
7	% Exposed relative to total amino acids – aspartic acid	0.00641
8	Molecular weight	0.00640
9	% Exposed relative to total amino acids - valine	0.00581
10	% Exposed relative to total exposed amino acids - threonine	0.00571

Table S3. Classifier predictions of high- vs. low-binding proteins on $(GT)_x$-SWCNTs.

Ranking	Protein	Accession Number	Probability
**$(GT)_{15}$-SWCNTs**			
**Ranking**	**Protein**	**Accession Number**	**Probability**
In 1	CD44 antigen	P16070	58.88%
In 2	Transgelin	Q01995	54.98%
In 3	TAR DNA-binding protein 43 (TDP-43)	Q13148	53.59%
Out 1	Lysozyme C	P00698	35.23%
Out 2	Ribonuclease pancreatic (RNase A)	P07998	32.91%
Out 3	Syntenin-1	O00560	30.13%
Out 4	L-lactate dehydrogenase A chain (LDH-A)	P00338	24.75%
Out 5	Glutathione S-transferase (GST)	Q8MU52	11.98%
**$(GT)_6$-SWCNTs**			
**Ranking**	**Protein**	**Accession Number**	**Probability**
In 1	TAR DNA-binding protein 43 (TDP-43)	Q13148	77.29%
In 2	Transgelin	Q01995	75.57%
In 3	CD44 antigen	P16070	60.38%
Out 1	Lysozyme C	P00698	47.77%
Out 2	Ribonuclease pancreatic (RNase A)	P07998	31.14%
Out 3	Syntenin-1	O00560	29.98%
Out 4	L-lactate dehydrogenase A chain (LDH-A)	P00338	15.94%
Out 5	Glutathione S-transferase (GST)	Q8MU52	12.91%

A simple kinetic model was fit to the ssDNA desorption data from the corona exchange assay:

$$\boldsymbol{D} + * \underset{\boldsymbol{k_2}}{\overset{\boldsymbol{k_1}}{\rightleftarrows}} \boldsymbol{D} * \qquad \textbf{Equation S1}$$

$$[*]_{\mathbf{tot}} = [*] + [\boldsymbol{D} *]$$

where D is ssDNA, $*$ is a SWCNT surface site, and $D*$ is ssDNA bound to a SWCNT surface site. Square brackets represent concentrations. Rate constants k_1, k_2, and total concentration of SWCNT surface sites $[*]_{\mathbf{tot}}$ were fit using an ordinary least-squares regression (**Fig. S7**).

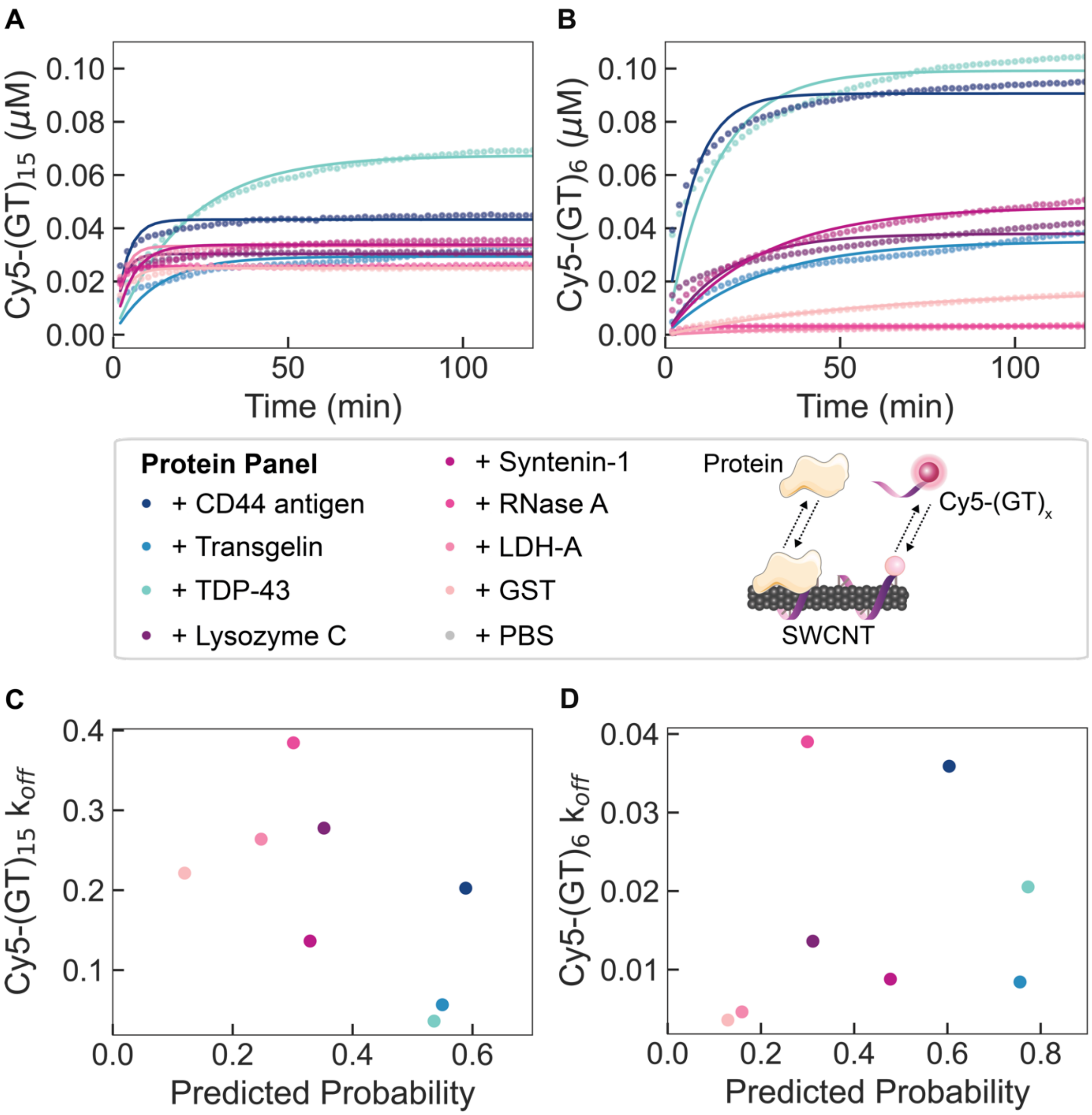

Fig. S7. Protein corona dynamics assessed for binding of predicted proteins to $(GT)_x$-SWCNTs. **(A-B)** A corona exchange assay determines binding of a protein panel (each at 80 mg L^{-1} final concentration) to **(A)** $(GT)_{15}$-SWCNTs or **(B)** $(GT)_6$-SWCNTs (each at 5 mg L^{-1} final concentration). ssDNA desorption from the SWCNT serves as a proxy for protein adsorption. Proteins are predicted by the RFC to be in the corona (probability > 0.5; blue-green colors) or out of the corona (probability < 0.5; purple-pink colors). The protein panel includes: CD44 antigen, transgelin, and TAR DNA-binding protein 43 (TDP-43) (predicted to be in the corona) and lysozyme C (Lys-C), syntenin-1, ribonuclease pancreatic (RNase A), L-lactate dehydrogenase A chain (LDH-A), and glutathione S-transferase (GST) (predicted to be out of the corona). Phosphate-buffered saline (PBS) is injected as a control and desorbed ssDNA is normalized to this initial value. Shaded error bars represent standard error between experimental replicates (N = 3).

(C-D) Rate constants of ssDNA desorption are compared to the RFC predicted in-corona probability for **(C)** $(GT)_{15}$-SWCNTs and **(D)** $(GT)_6$-SWCNTs.

Data S1. (separate file)
MS Proteomic Datasheet SI

REFERENCES AND NOTES

1. L. Gloag, M. Mehdipour, D. Chen, R. D. Tilley, J. J. Gooding, Advances in the application of magnetic nanoparticles for sensing. *Adv. Mater.* **31**, 1904385 (2019).

2. P. D. Howes, R. Chandrawati, M. M. Stevens, Colloidal nanoparticles as advanced biological sensors. *Science* **346**, 1247390 (2014).

3. J. T. Del Bonis-O'Donnell, L. Chio, G. F. Dorlhiac, I. R. McFarlane, M. P. Landry, Advances in nanomaterials for brain microscopy. *Nano Res.* **11**, 5144–5172 (2018).

4. B. R. Smith, S. S. Gambhir, Nanomaterials for in vivo imaging. *Chem. Rev.* **117**, 901–986 (2017).

5. M. J. Mitchell, M. M. Billingsley, R. M. Haley, M. E. Wechsler, N. A. Peppas, R. Langer, Engineering precision nanoparticles for drug delivery. *Nat. Rev. Drug Discov.* **20**, 101–124 (2021).

6. J. W. Wang, E. G. Grandio, G. M. Newkirk, G. S. Demirer, S. Butrus, J. P. Giraldo, M. P. Landry, Nanoparticle-mediated genetic engineering of plants. *Mol. Plant* **12**, 1037–1040 (2019).

7. W. Poon, B. R. Kingston, B. Ouyang, W. Ngo, W. C. W. Chan, A framework for designing delivery systems. *Nat. Nanotechnol.* **15**, 819–829 (2020).

8. G. Hong, S. Diao, J. Chang, A. L. Antaris, C. Chen, B. Zhang, S. Zhao, D. N. Atochin, P. L. Huang, K. I. Andreasson, C. J. Kuo, H. Dai, Through-skull fluorescence imaging of the brain in a new near-infrared window. *Nat. Photonics* **8**, 723–730 (2014).

9. O. T. Bruns, T. S. Bischof, D. K. Harris, D. Franke, Y. Shi, L. Riedemann, A. Bartelt, F. B. Jaworski, J. A. Carr, C. J. Rowlands, M. W. B. Wilson, O. Chen, H. Wei, G. W. Hwang, D. M. Montana, I. Coropceanu, O. B. Achorn, J. Kloepper, J. Heeren, P. T. C. So, D. Fukumura, K. F. Jensen, R. K. Jain, M. G. Bawendi, Next-generation in vivo optical imaging with short-wave infrared quantum dots. *Nat. Biomed. Eng.* **1**, 0056 (2017).

10. H. Safari, N. Kaczorowski, M. L. Felder, E. R. Brannon, M. Varghese, K. Singer, O. Eniola-Adefeso, Biodegradable, bile salt microparticles for localized fat dissolution. *Sci. Adv.* **6**, eabd8019 (2020).

11. R. L. Ball, K. A. Hajj, J. Vizelman, P. Bajaj, K. A. Whitehead, Lipid nanoparticle formulations for enhanced co-delivery of siRNA and mRNA. *Nano Lett.* **18**, 3814–3822 (2018).

12. L. Xiao, G. Lu, Q. Lu, D. L. Kaplan, Direct formation of silk nanoparticles for drug delivery. *ACS Biomater Sci. Eng.* **2**, 2050–2057 (2016).

13. A. J. Gillen, A. A. Boghossian, Non-covalent methods of engineering optical sensors based on single-walled carbon nanotubes. *Front. Chem.* **7**, 612 (2019).

14. A. A. Boghossian, J. Zhang, P. W. Barone, N. F. Reuel, J.-H. Kim, D. A. Heller, J.-H. Ahn, A. J. Hilmer, A. Rwei, J. R. Arkalgud, C. T. Zhang, M. S. Strano, Near-infrared fluorescent sensors based on single-walled carbon nanotubes for life sciences applications. *ChemSusChem* **4**, 848–863 (2011).

15. Z. Liu, S. Tabakman, K. Welsher, H. Dai, Carbon nanotubes in biology and medicine: In vitro and in vivo detection, imaging and drug delivery. *Nano Res.* **2**, 85–120 (2009).

16. S. Kruss, M. P. Landry, E. Vander Ende, B. M. A. Lima, N. F. Reuel, J. Zhang, J. Nelson, B. Mu, A. Hilmer, M. Strano, Neurotransmitter detection using corona phase molecular recognition on fluorescent single-walled carbon nanotube sensors. *J. Am. Chem. Soc.* **136**, 713–724 (2014).

17. A. G. Beyene, K. Delevich, J. T. D. Bonis-O'Donnell, D. J. Piekarski, W. C. Lin, A. W. Thomas, S. J. Yang, P. Kosillo, D. Yang, G. S. Prounis, L. Wilbrecht, M. P. Landry, Imaging striatal dopamine release using a nongenetically encoded near infrared fluorescent catecholamine nanosensor. *Sci. Adv.* **5**, eaaw3108 (2019).

18. S. Jeong, D. Yang, A. G. Beyene, J. T. D. Bonis-O'Donnell, A. M. M. Gest, N. Navarro, X. Sun, M. P. Landry, High-throughput evolution of near-infrared serotonin nanosensors. *Sci. Adv.* **5**, eaay3771 (2019).

19. L. Chio, J. T. Del Bonis-O'Donnell, M. A. Kline, J. H. Kim, I. R. McFarlane, R. N. Zuckermann, M. P. Landry, Electrostatic assemblies of single-walled carbon nanotubes and sequence-tunable peptoid polymers detect a lectin protein and its target sugars. *Nano Lett.* **19**, 7563–7572 (2019).

20. R. L. Pinals, F. Ledesma, D. Yang, N. Navarro, S. Jeong, J. E. Pak, L. Kuo, Y.-C. Chuang, Y.-W. Cheng, H.-Y. Sun, M. P. Landry, Rapid SARS-CoV-2 spike protein detection by carbon nanotube-based near-infrared nanosensors. *Nano Lett.* **21**, 2272–2280 (2021).

21. G. S. Demirer, H. Zhang, J. L. Matos, N. S. Goh, F. J. Cunningham, Y. Sung, R. Chang, A. J. Aditham, L. Chio, M.-J. Cho, B. Staskawicz, M. P. Landry, High aspect ratio nanomaterials enable delivery of functional genetic material without DNA integration in mature plants. *Nat. Nanotechnol.* **14**, 456–464 (2019).

22. G. S. Demirer, H. Zhang, N. S. Goh, R. L. Pinals, R. Chang, M. P. Landry, Carbon nanocarriers deliver siRNA to intact plant cells for efficient gene knockdown. *Sci. Adv.* **6**, eaaz0495 (2020).

23. M. P. Monopoli, C. Åberg, A. Salvati, K. A. Dawson, Biomolecular coronas provide the biological identity of nanosized materials. *Nat. Nanotechnol.* **7**, 779–786 (2012).

24. A. E. Nel, L. Mädler, D. Velegol, T. Xia, E. M. V. Hoek, P. Somasundaran, F. Klaessig, V. Castranova, M. Thompson, Understanding biophysicochemical interactions at the nano–bio interface. *Nat. Mater.* **8**, 543–557 (2009).

25. P. C. Ke, S. Lin, W. J. Parak, T. P. Davis, F. Caruso, A decade of the protein corona. *ACS Nano* **11**, 11773–11776 (2017).

26. C. Jiang, G. Wang, R. Hein, N. Liu, X. Luo, J. J. Davis, Antifouling strategies for selective in vitro and in vivo sensing. *Chem. Rev.* **120**, 3852–3889 (2020).

27. D. Yang, S. J. Yang, J. T. Del Bonis-O’Donnell, R. L. Pinals, M. P. Landry, Mitigation of carbon nanotube neurosensor induced transcriptomic and morphological changes in mouse microglia with surface passivation. *ACS Nano* **14**, 13794–13805 (2020).

28. R. L. Pinals, D. Yang, A. Lui, W. Cao, M. P. Landry, Corona exchange dynamics on carbon nanotubes by multiplexed fluorescence monitoring. *J. Am. Chem. Soc.* **142**, 1254–1264 (2020).

29. Q. Dai, N. Bertleff-Zieschang, J. A. Braunger, M. Björnmalm, C. Cortez-Jugo, F. Caruso, Particle targeting in complex biological media. *Adv. Healthc. Mater.* **7**, 1700575 (2018).

30. P. S. R. Naidu, N. Gavriel, C. G. G. Gray, C. A. Bartlett, L. M. Toomey, J. A. Kretzmann, D. Patalwala, T. McGonigle, E. Denham, C. Hee, D. Ho, N. L. Taylor, M. Norret, N. M. Smith, S. A. Dunlop, K. S. Iyer, M. Fitzgerald, Elucidating the inability of functionalized nanoparticles to cross the blood–brain barrier and target specific cells in vivo. *ACS Appl. Mater. Interfaces* **11**, 22085–22095 (2019).

31. M. Mahmoudi, Debugging nano–bio interfaces: Systematic strategies to accelerate clinical translation of nanotechnologies. *Trends Biotechnol.* **36**, 755–769 (2018).

32. H. He, L. Liu, E. E. Morin, M. Liu, A. Schwendeman, Survey of clinical translation of cancer nanomedicines—Lessons learned from successes and failures. *Acc. Chem. Res.* **52**, 2445–2461 (2019).

33. R. L. Pinals, L. Chio, F. Ledesma, M. P. Landry, Engineering at the nano-bio interface: Harnessing the protein corona towards nanoparticle design and function. *Analyst* **145**, 5090–5112 (2020).

34. N. Bertrand, P. Grenier, M. Mahmoudi, E. M. Lima, E. A. Appel, F. Dormont, J.-M. Lim, R. Karnik, R. Langer, O. C. Farokhzad, Mechanistic understanding of in vivo protein corona formation on polymeric nanoparticles and impact on pharmacokinetics. *Nat. Commun.* **8**, 1–8 (2017).

35. K. A. Dawson, Y. Yan, Current understanding of biological identity at the nanoscale and future prospects. *Nat. Nanotechnol.* **16**, 229–242 (2021).

36. J. Lazarovits, S. Sindhwani, A. J. Tavares, Y. Zhang, F. Song, J. Audet, J. R. Krieger, A. M. Syed, B. Stordy, W. C. W. Chan, Supervised learning and mass spectrometry predicts the in vivo fate of nanomaterials. *ACS Nano* **13**, 8023–8034 (2019).

37. K. M. Poulsen, T. Pho, J. A. Champion, C. K. Payne, Automation and low-cost proteomics for characterization of the protein corona: Experimental methods for big data. *Anal. Bioanal. Chem.* **412**, 6543–6551 (2020).

38. R. Oliverio, B. Liberelle, F. Murschel, A. Garcia-Ac, X. Banquy, G. De Crescenzo, Versatile and high-throughput strategy for the quantification of proteins bound to nanoparticles. *ACS Appl. Nano Mater.* **3**, 10497–10507 (2020).

39. M. R. Findlay, D. N. Freitas, M. Mobed-Miremadi, K. E. Wheeler, Machine learning provides predictive analysis into silver nanoparticle protein corona formation from physicochemical properties. *Environ. Sci. Nano* **5**, 64–71 (2018).

40. C. D. Walkey, J. B. Olsen, F. Song, R. Liu, H. Guo, D. W. H. Olsen, Y. Cohen, A. Emili, W. C. W. Chan, Protein corona fingerprinting predicts the cellular interaction of gold and silver nanoparticles. *ACS Nano* **8**, 2439–2455 (2014).

41. Z. Ban, P. Yuan, F. Yu, T. Peng, Q. Zhou, X. Hu, Machine learning predicts the functional composition of the protein corona and the cellular recognition of nanoparticles. *Proc. Natl. Acad. Sci. U.S.A.* **117**, 10492–10499 (2020).

42. Y. Duan, R. Coreas, Y. Liu, D. Bitounis, Z. Zhang, D. Parviz, M. Strano, P. Demokritou, W. Zhong, Prediction of protein corona on nanomaterials by machine learning using novel descriptors. *NanoImpact* **17**, 100207 (2020).

43. D. Fourches, D. Pu, C. Tassa, R. Weissleder, S. Y. Shaw, R. J. Mumper, A. Tropsha, Quantitative nanostructure−activity relationship modeling. *ACS Nano* **4**, 5703–5712 (2010).

44. X. Bai, F. Liu, Y. Liu, C. Li, S. Wang, H. Zhou, W. Wang, H. Zhu, D. A. Winkler, B. Yan, Toward a systematic exploration of nano-bio interactions. *Toxicol. Appl. Pharmacol.* **323**, 66–73 (2017).

45. G. Yamankurt, E. J. Berns, A. Xue, A. Lee, N. Bagheri, M. Mrksich, C. A. Mirkin, Exploration of the nanomedicine-design space with high-throughput screening and machine learning. *Nat. Biomed. Eng.* **3**, 318–327 (2019).

46. A. Antonucci, J. Kupis-Rozmysłowicz, A. A. Boghossian, Noncovalent protein and peptide functionalization of single-walled carbon nanotubes for biodelivery and optical sensing applications. *ACS Appl. Mater. Interfaces* **9**, 11321–11331 (2017).

47. M. Di Giosia, F. Valle, A. Cantelli, A. Bottoni, F. Zerbetto, E. Fasoli, M. Calvaresi, Identification and preparation of stable water dispersions of protein - Carbon nanotube hybrids and efficient design of new functional materials. *Carbon* **147**, 70–82 (2019).

48. R. L. Pinals, D. Yang, D. J. Rosenberg, T. Chaudhary, A. R. Crothers, A. T. Iavarone, M. Hammel, M. P. Landry, Quantitative protein corona composition and dynamics on carbon nanotubes in biological environments. *Angew. Chem. Int. Ed.* **59**, 23668–23677 (2020).

49. The UniProt Consortium, UniProt: The universal protein knowledgebase in 2021. *Nucleic Acids Res.* **49**, D480–D489 (2021).

50. P. J. A. Cock, T. Antao, J. T. Chang, B. A. Chapman, C. J. Cox, A. Dalke, I. Friedberg, T. Hamelryck, F. Kauff, B. Wilczynski, M. J. L. de Hoon, Biopython: Freely available Python tools for computational molecular biology and bioinformatics. *Bioinformatics* **25**, 1422–1423 (2009).

51. M. S. Klausen, M. C. Jespersen, H. Nielsen, K. K. Jensen, V. I. Jurtz, C. K. Sønderby, M. O. A. Sommer, O. Winther, M. Nielsen, B. Petersen, P. Marcatili, NetSurfP-2.0: Improved prediction of protein structural features by integrated deep learning. *Proteins Struct. Funct. Genet.* **87**, 520–527 (2019).

52. L. Breiman, Random forests. *Mach. Learn.* **45**, 5–32 (2001).

53. N. V. Chawla, K. W. Bowyer, L. O. Hall, W. P. Kegelmeyer, SMOTE: Synthetic minority over-sampling technique. *J. Artif. Intell. Res.* **16**, 321–357 (2002).

54. M. Gravely, M. M. Safaee, D. Roxbury, Biomolecular functionalization of a nanomaterial to control stability and retention within live cells. *Nano Lett.* **19**, 6203–6212 (2019).

55. S. Wang, E. S. Humphreys, S.-Y. Chung, D. F. Delduco, S. R. Lustig, H. Wang, K. N. Parker, N. W. Rizzo, S. Subramoney, Y.-M. Chiang, A. Jagota, Peptides with selective affinity for carbon nanotubes. *Nat. Mater.* **2**, 196–200 (2003).

56. J. Liu, L. Yang, A. J. Hopfinger, Affinity of drugs and small biologically active molecules to carbon nanotubes: A pharmacodynamics and nanotoxicity factor? *Mol. Pharm.* **6**, 873–882 (2009).

57. M. Vihinen, E. Torkkila, P. Riikonen, Accuracy of protein flexibility predictions. *Proteins* **19**, 141–149 (1994).

58. M. Saeedimasine, E. G. Brandt, A. P. Lyubartsev, Atomistic perspective on biomolecular adsorption on functionalized carbon nanomaterials under ambient conditions. *J. Phys. Chem. B* **125**, 416–430 (2021).

59. H. Chaudhary, R. M. F. Fernandes, V. Gowda, M. M. A. E. Claessens, I. Furó, C. Lendel, Intrinsically disordered protein as carbon nanotube dispersant: How dynamic interactions lead to excellent colloidal stability. *J. Colloid Interface Sci.* **556**, 172–179 (2019).

60. A. A. Alizadehmojarad, X. Zhou, A. G. Beyene, K. E. Chacon, Y. Sung, R. L. Pinals, M. P. Landry, L. Vuković, Binding affinity and conformational preferences influence kinetic stability of short oligonucleotides on carbon nanotubes. *Adv. Mater. Interfaces* **7**, 2000353 (2020).

61. J. F. Campbell, I. Tessmer, H. H. Thorp, D. A. Erie, Atomic force microscopy studies of DNA-wrapped carbon nanotube structure and binding to quantum dots. *J. Am. Chem. Soc.* **130**, 10648–10655 (2008).

62. A. G. Beyene, A. A. Alizadehmojarad, G. Dorlhiac, N. Goh, A. M. Streets, P. Král, L. Vuković, M. P. Landry, Ultralarge modulation of fluorescence by neuromodulators in carbon nanotubes functionalized with self-assembled oligonucleotide rings. *Nano Lett.* **18**, 6995–7003 (2018).

63. D. Roxbury, J. Mittal, A. Jagota, Molecular-basis of single-walled carbon nanotube recognition by single-stranded DNA. *Nano Lett.* **12**, 1464–1469 (2012).

64. F. Schöppler, C. Mann, T. C. Hain, F. M. Neubauer, G. Privitera, F. Bonaccorso, D. Chu, A. C. Ferrari, T. Hertel, Molar extinction coefficient of single-wall carbon nanotubes. *J. Phys. Chem. C* **115**, 14682–14686 (2011).

65. F. K. Brunecker, F. Schöppler, T. Hertel, Interaction of polymers with single-wall carbon nanotubes. *J. Phys. Chem. C* **120**, 10094–10103 (2016).

66. A. Hirano, T. Kameda, Aromaphilicity index of amino acids: Molecular dynamics simulations of the protein binding affinity for carbon nanomaterials. *ACS Appl. Nano Mater.* **4**, 2486–2495 (2021).

67. Z. He, J. Zhou, Probing carbon nanotube–amino acid interactions in aqueous solution with molecular dynamics simulations. *Carbon* **78**, 500–509 (2014).

68. V. Zorbas, A. L. Smith, H. Xie, A. Ortiz-Acevedo, A. B. Dalton, G. R. Dieckmann, R. K. Draper, R. H. Baughman, I. H. Musselman, Importance of aromatic content for peptide/single-walled carbon nanotube interactions. *J. Am. Chem. Soc.* **127**, 12323–12328 (2005).

69. S. M. Tomásio, T. R. Walsh, Modeling the binding affinity of peptides for graphitic surfaces. Influences of aromatic content and interfacial shape. *J. Phys. Chem. C* **113**, 8778–8785 (2009).

70. T. A. Davis, L. A. Holland, Peptide probe for multiwalled carbon nanotubes: Electrophoretic assessment of the binding interface and evaluation of surface functionalization. *ACS Appl. Mater. Interfaces* **10**, 11311–11318 (2018).

71. D. Nepal, K. E. Geckeler, pH-sensitive dispersion and debundling of single-walled carbon nanotubes: Lysozyme as a tool. *Small* **2**, 406–412 (2006).

72. D. W. Horn, K. Tracy, C. J. Easley, V. A. Davis, Lysozyme dispersed single-walled carbon nanotubes: Interaction and activity. *J. Phys. Chem. C* **116**, 10341–10348 (2012).

73. B. D. Holt, M. C. McCorry, P. D. Boyer, K. N. Dahl, M. F. Islam, Not all protein-mediated single-wall carbon nanotube dispersions are equally bioactive. *Nanoscale* **4**, 7425–7434 (2012).

74. K. Matsuura, T. Saito, T. Okazaki, S. Ohshima, M. Yumura, S. Iijima, Selectivity of water-soluble proteins in single-walled carbon nanotube dispersions. *Chem. Phys. Lett.* **429**, 497–502 (2006).

75. M. Zöller, CD44: Can a cancer-initiating cell profit from an abundantly expressed molecule? *Nat. Rev. Cancer* **11**, 254–267 (2011).

76. A. Rives, J. Meier, T. Sercu, S. Goyal, Z. Lin, J. Liu, D. Guo, M. Ott, C. L. Zitnick, J. Ma, R. Fergus, Biological structure and function emerge from scaling unsupervised learning to 250 million protein sequences. *Proc. Natl. Acad. Sci. U.S.A.* **118**, e2016239118 (2021).

77. F. Pedregosa, G. Varoquaux, A. Gramfort, V. Michel, B. Thirion, O. Grisel, M. Blondel, P. Prettenhofer, R. Weiss, V. Dubourg, J. Vanderplas, A. Passos, D. Cournapeau, Scikit-learn: Machine learning in Python. *J. Mach. Learn. Res.* **12**, 2825–2830 (2011).

78. N. Ouassil,* R. L. Pinals,* J. T. Del Bonis-O'Donnell, J. W. Wang, M. P. Landry, Supervised learning model predicts protein adsorption to carbon nanotubes (version 0.0.1). Zenodo (2021), doi.org/10.5281/zenodo.5640140 [Computer Software]; doi:10.5281/zenodo.5641450 [Data].

79. N. Otsu, A threshold selection method from gray-level histograms. *IEEE Trans. Syst. Man Cybern.* **9**, 62–66 (1979).

80. T. Chen, C. Guestrin, XGBoost: A scalable tree boosting system, in *KDD '16: Proceedings of the 22nd ACM SIGKDD International Conference on Knowledge Discovery and Data Mining* (Association for Computing Machinery, 2016), pp. 785–794; https://doi.org/10.1145/2939672.2939785.

81. D. Roxbury, P. V. Jena, Y. Shamay, C. P. Horoszko, D. A. Heller, Cell membrane proteins modulate the carbon nanotube optical bandgap via surface charge accumulation. *ACS Nano* **10**, 499–506 (2016).

82. M. Abadi, P. Barham, J. Chen, Z. Chen, A. Davis, J. Dean, M. Devin, S. Ghemawat, G. Irving, M. Isard, M. Kudlur, J. Levenberg, R. Monga, S. Moore, D.G. Murray, B. Steiner, P. Tucker, V. Vasudevan, P. Warden, M. Wicke, Y. Yu, X. Zheng, TensorFlow: A system for large-scale machine learning, in *12th USENIX Symposium on Operating Systems Design and Implementation (OSDI 16)* (USENIX Association, 2016), pp. 265–283.

83. J. Kyte, R. F. Doolittle, A simple method for displaying the hydropathic character of a protein. *J. Mol. Biol.* **157**, 105–132 (1982).
